# How a Green Roof Becomes Biodiverse: Vegetation Analysis on a Green Roof with no Maintenance in Rome (Italy)

**DOI:** 10.3390/plants14203180

**Published:** 2025-10-16

**Authors:** Amii Bellini, Valentina Savo, Giulia Caneva, Elettra D’Amico, Roberto Casalini, Flavia Bartoli

**Affiliations:** 1 Department of Science, Roma Tre University, Viale Marconi 446, 00146 Rome, Italy; amii.bellini@uniroma3.it (A.B.); giulia.caneva@uniroma3.it (G.C.); ele.damico1@stud.uniroma3.it (E.D.); flavia.bartoli@uniroma3.it (F.B.); 2 Department of Education Science, Roma Tre University, Via del Castro Pretorio 20, 00185 Rome, Italy; 3 National Biodiversity Future Center (NBFC), University of Palermo, Piazza Marina 61, 90133 Palermo, Italy; 4 Liceo Scientifico Statale Giovanni Keplero, Via Silvestro Gherardi, 87, 00146 Rome, Italy; casalini.roberto@liceokepleroroma.edu.it; 5 Institute of Heritage Science (CNR-ISPC), National Research Council of Italy, Area della Ricerca di Roma 1, Strada della Neve s.n.c., 00010 Montelibretti, Italy

**Keywords:** urban biodiversity, natural colonization, Mediterranean green roofs, urban living roofs

## Abstract

Green roofs have increasingly been used in urban contexts to increase thermal insulation, provide habitat for species, and increase urban biodiversity. Here, we provide the results of a monitoring experiment to document (1) the survival rate of planted species of a green roof with no maintenance and (2) the natural colonization by new species of the same roof. Each month for one year, we conducted floristic and vegetation surveys, identifying the species of colonizers and monitoring the cover of both planted and wild species. We conducted various statistical tests to determine the driving factors of spontaneous plants’ colonization of the unattended green roof. Among the planted species, several Mediterranean species thrived despite the lack of irrigation, and among these, *Thymus serpyllum* L. (a prostrate shrub) maintained the highest cover. The spontaneous colonization involved 62 species, including Mediterranean (38%) and exotic species (15%), primarily annual ruderals. The difficult climatic and pedological conditions (i.e., solar irradiation, soil structure) of the green roof have driven the colonization process and the survival of the colonizers. Research on dynamic colonization processes can contribute to designing green roofs with greater biodiversity, a more sustainable approach to long-term management, enhanced urban climate adaptation, and greater aesthetic appeal.

## 1. Introduction

Urban green infrastructure represents a crucial solution for improving urban resilience and livability in an increasingly urbanized world [1]. Among these solutions, green roofs offer a unique opportunity to enhance urban biodiversity and ecosystem functionality by utilizing otherwise unused space [2]. These systems provide multiple ecosystem services through interrelated mechanisms that depend on their design and biological components [3]. To increase these benefits, there has been a significant increment in experiments and investigations aimed at integrating green roofs within the urban environment [4].

While initially implemented to improve building energy efficiency through enhanced thermal performance [5,6], green roofs have evolved to deliver broader benefits, including improved air quality and reduced heat island effects [7,8,9,10,11]. When properly designed, they can create vital habitats for urban wildlife [12,13,14,15,16]. Native plants are particularly valuable as they are adapted to local conditions, resist local pests, and support indigenous fauna [6,15]. Additionally, higher plant diversity directly enhances roof functionality through complementary traits and resource utilization patterns, resulting in more efficient delivery of ecosystem services. Species diversity and structural heterogeneity improve stormwater retention [17], enhance thermal regulation [18], increase pollution capture [19], and expand habitat availability [20], while simultaneously enhancing aesthetic value [21]. However, current practices often limit plant selection to *Sedum* or other succulent species [22,23], citing challenging growing conditions [24]. Research has suggested that native species could effectively complement or even replace *Sedum* carpets [25,26,27], creating more diverse and ecologically valuable systems.

Plants tend to find suitable habitats, and even in engineered ecosystems such as extensive green roofs, natural (re)colonization may occur (e.g., [28,29,30,31,32]). Several studies have suggested that naturally occurring native species could increase green roofs’ biodiversity and ecological value [22,25,26,27,33,34,35,36,37]. Biodiverse green roofs can resemble natural habitats with a certain diversity of native species [28]. *Sedum* or other succulent species can withstand the stressful conditions of extensive green roofs [27]; however, they are generally used as low-diversity communities that provide ecosystem services to a lesser extent [17,38].

Green roof design traditionally varies between intensive systems with deep soil layers resembling ground gardens and extensive systems featuring shallow substrates and low-growing vegetation [2]. While the implementation of extensive green roofs is increasing in many countries worldwide, the Mediterranean climatic and environmental conditions can pose several constraints on plant growth and development, particularly the severe summer droughts and high solar irradiation [39,40,41,42]. For this reason, in southern Mediterranean countries, the implementation of extensive green roofs is not as common as in cities in central or northern Europe [26,43]. Therefore, research on these green roofs is relatively limited [41], while studies analyzing colonization processes on Mediterranean green roofs are almost nonexistent. Several studies have addressed the energy or hydraulic performance of green roofs in Mediterranean areas [44,45,46], while fewer have analyzed the plant ecological features and dynamics [47,48] of these ecosystems. A limited number of studies have analyzed the performance of native plants on green roofs in the Mediterranean area [48,49,50,51,52]. This research is timely, given the increasing urbanization in the Mediterranean region and the exacerbating effects of climate change, which are projected to intensify drought and heat stress in the area [53,54]. First, it can provide crucial information for designing low-maintenance, high-resilience systems, which are particularly pivotal in addressing water and heat stress [38,55]. Second, identifying spontaneous species adapted to the extreme conditions of Mediterranean green roofs can significantly reduce installation and maintenance costs, potentially increasing the diffusion of these green solutions in Mediterranean urban areas [18,25]. Third, understanding ecological succession patterns in these artificial environments can contribute to urban biodiversity conservation, creating new habitats for urban species [56,57,58].

Here, we present a case study of the natural recolonization of an extensive green roof with no maintenance in Rome (Italy), hypothesizing that some plants could grow despite human intervention. We performed a one-year monitoring experiment, through monthly vegetation surveys, and analyzed (1) how the initially planted species reacted to the lack of watering, identifying potential candidates for low-maintenance green roofs in a Mediterranean climate; (2) the spontaneous colonization process of the green roof by naturally occurring plant species, assessing their potential contribution to the resilience and biodiversity of these systems. We also analyzed vegetation changes to understand the potential influence of environmental conditions on the growth of planted and colonizing species. The results of this study offer indications for designing more sustainable and economically viable green roofs in Mediterranean climates.

## 2. Results

### 2.1. Evaluation of the Survival Rates of Planted Species Without Irrigation

**Figure 1:**
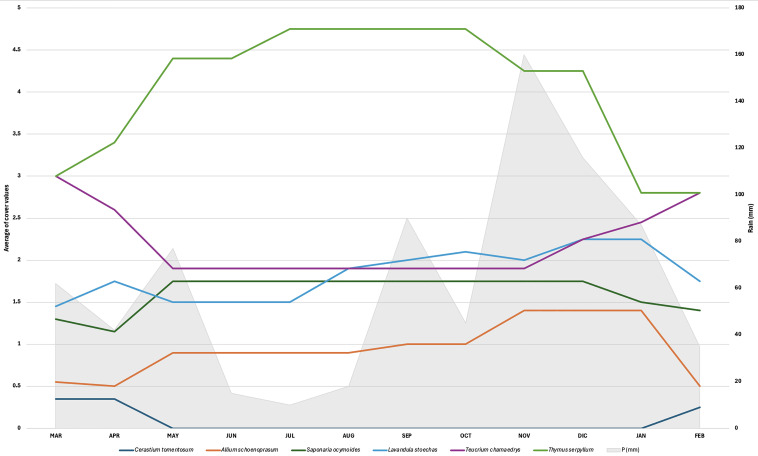
Monthly change in the average cover values of the cultivated species from March 2021 to February 2022. We transformed the vegetation cover data (percentage cover) from the Braun–Blanquet scale to the Van der Mareel scale. We then added the obtained numerical values for each species and calculated the average of the cover values of the sections. In the back (the shadowed area in gray), we show the precipitation data for the same period.

We evaluated the survival rate of the planted species across all sampling areas: the more exposed section of the extensive green roof (E), the more sheltered area of the extensive green roof (S), and the six raised garden bed planters with a thicker growing substrate (C1–C6). The graph in Figure 1 shows each species’ overall growth trend and cover changes across all sampling areas from March 2021 to February 2022, representing the average cover values across all sampling areas where each species was present. *Thymus serpyllum* showed steady growth and cover across the areas and months, with a more extensive expansion over the summer (between July and September), consistent with its prostrate growth habit that naturally tends to form dense carpets. *Teucrium chamaedrys, Lavandula stoechas*, and *Saponaria ocymoides* performed well over the year (i.e., their cover values did not change dramatically over the analyzed period). *Cerastium tomentosum* had the lowest cover values. *Allium schoenoprasum* (growing in the raised garden bed planters C1, C3, C5, and on the roof surface E), despite its morphology that inherently limits potential coverage values, maintained its cover values over the year. Detailed monthly observations for each species and sampling area are provided in the Supplementary Files (Tables S1–S8).

### 2.2. Evaluation of the Natural Recolonization Dynamics

**Figure 2:**
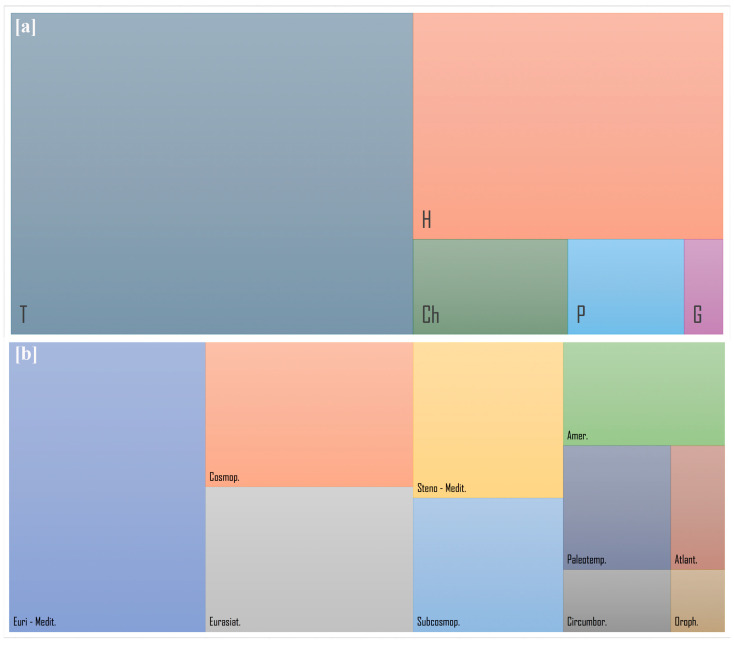
Treemap chart of the (**a**) Life form and (**b**) Chorotype of the colonizing plants. Annual Mediterranean plants are predominant. Notes: T = Therophytes; H = Hemicryptophytes; P = Phanerophytes; Ch = Chamaephytes; G = Geophytes; Euromedit. = Euromediterranean; Cosmop. = Cosmopolitan; Subcosmop. = Sub cosmopolitan; Eurasiat. = Eurasiatic; Paleotemp. = Paleotemperate; Stenomedit = Stenomediterranean; Amer. = American; Atlant. = Atlantic; Circumbor. = Circumboreal; Oroph. = Orophyte.

The analysis of the vegetation patterns revealed some differences in environmental conditions among the various sections of the green roof. Between March 2021 and February 2022, we observed 62 colonizing species belonging to 18 families with different chorotypes and biological forms. Many species (19 species, 31%) were of the Asteraceae family, and several others (10 species, 16%) of the Poaceae family (Supplementary Table S9). The analysis of the life forms and chorotypes showed that the colonists fit the ecological conditions of the roof (Figure 2a,b). Among the colonizing species, therophytes were the most abundant (35 species, 56%), hemicryptophytes were 19 (31%), chamaephytes were four (6%), phanerophytes were three (5%), and there was only one geophyte (2%). In the raised garden bed planters, there were more Hemicryptophytes compared to the other areas. Figure 2b shows the distribution of the plant chorotypes. Most species are Mediterranean (27% of the species belong to the Euri-Mediterranean chorotype, while 11% are Steno-Mediterranean). Many plants (25% of the species) are also Cosmopolitan or Subcosmopolitan.

The vegetation surveys (see Supplementary Files, Tables S10–S17) showed a predominance of the *Stellarietea mediae* class (26 species), which groups short-lived nitrophilous and semi-nitrophilous weeds. Species within this class generally have a wide geographical distribution. Within this class, the sub-class *Chenopodio-Stellarienea* (13 species) was quite well represented; this sub-class includes short-lived synanthropic nitrophilous and semi-nitrophilous weeds that grow in ruderal habitats. Another well-represented class was the *Polygono arenastri-Poetea annuae* (5 species) and the *Parietarietea judaicae* (four species), which includes annual (Therophytes) and perennial (Hemicryptophytes) synanthropic nitrophilous species that grow on impervious surfaces and on brick and rock walls.

**Figure 3:**
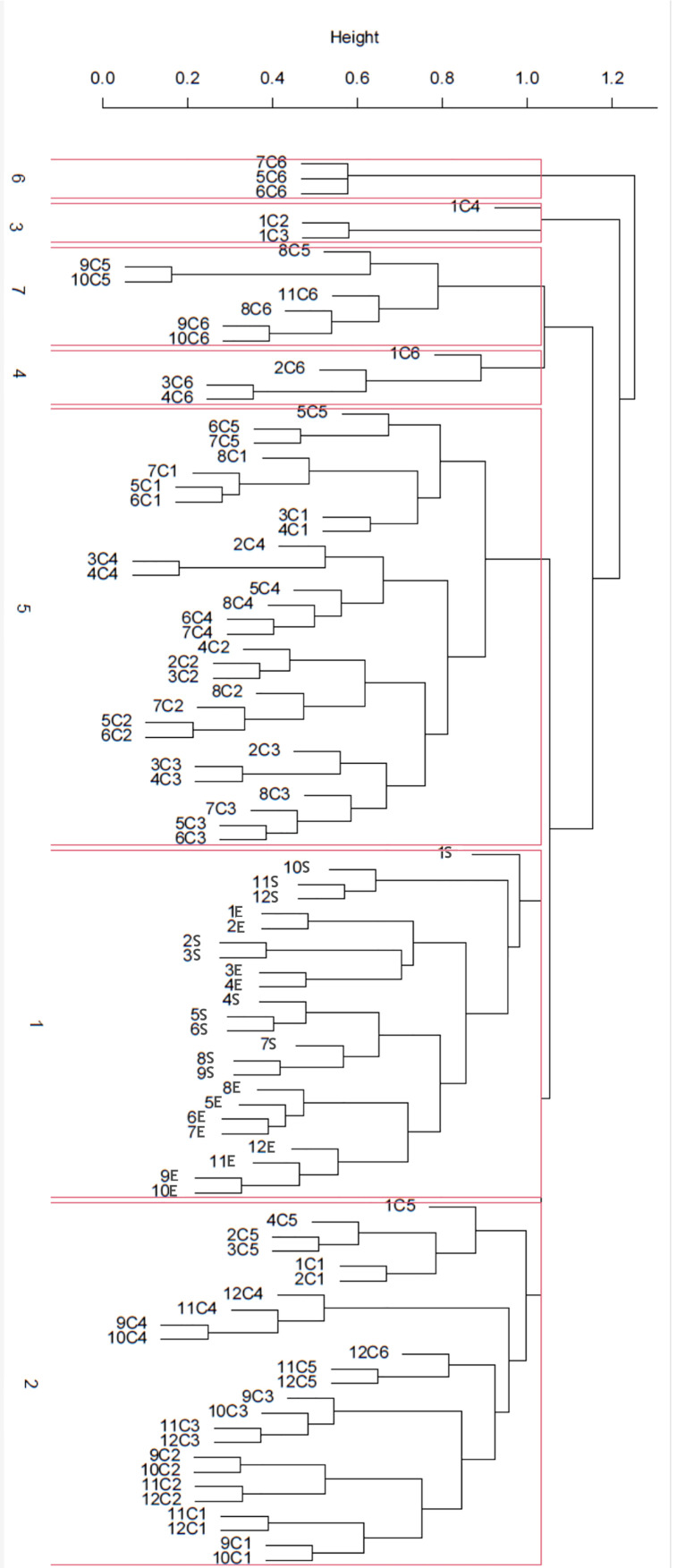
UPGMA Cluster Analysis. The seven clusters, separated by a red line, define the changes in the vegetation communities on the green roof identified by the analysis. This means that each sampled (spatiotemporal) community is more similar to other communities within the same group than any other community. Notes: the first number indicates the sampling month (i.e., 1 stands for January, 2 for February, etc.); E stands for the exposed area, while S stands for the sheltered part of the green roof; C1, C2, C3, C4, C5, C6 are the six raised garden bed planters.

Figure 3 shows the results of the cluster analysis using the UPMGA (Unweighted Arithmetic Average Clustering, [59]) method. This analysis divided the plant community into seven clusters according to differences in floristic composition and vegetation dynamics. Based on the structure of the cluster as a whole, we carried out a separation at level one, thus obtaining seven main clusters. The extensive sections of the Keplero High School (E and S) are grouped in a single cluster (Cluster 1), which does not include the surveys carried out in the raised garden bed planters (C1–C6). This clear separation highlights the importance of edaphic differences and soil thickness in determining different floristic patterns. Cluster 2 brings together the raised planters C1 and C5, while Cluster 5 brings together mainly the raised planters C2, C3, and C4, which therefore show a certain similarity with each other. From this analysis, it emerges that the C6 raised planter is the one that separates most clearly from the others. Also, the periodic surveys in the same sampling areas tend to group together. This possibly indicates that the environmental differences in the various raised garden bed planters (ventilation, shading, etc.) had greater weight than the differences deriving from the temporal succession of plant communities growing in the planters.

**Figure 4:**
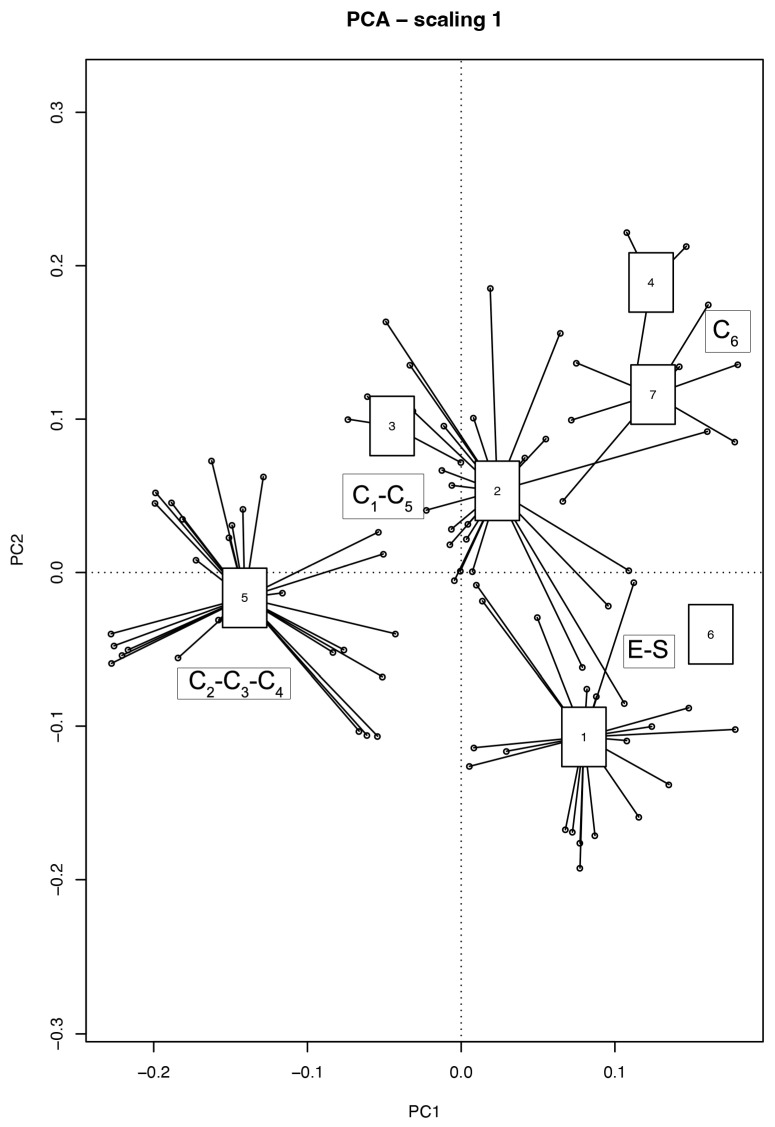
The Principal Component Analysis (PCA) of the seven clusters (see Figure 3). This analysis shows the ecological affinity/diversity of the species among the surveyed plant communities. Notes: Numbers correspond to the cluster group numbers. Cluster number 1 includes the vegetation surveys on the E and S sections of the green roof.

The Principal Component Analysis (PCA) (Figure 4) outlined the ecological differences among the clusters. Following axis one, from left to right, we observe, first of all the sampling areas C2, C3, and C4, then C1, C5, and C6, while, on the right side of the graph, we observe the S and O sampling areas, very close to each other, yet distant from the rest of the groups. This distancing confirms the influence of soil thickness as the first limiting factor. Furthermore, we note that C4 and C6 are separated from each other, and C3 is distant from C1. The separation between C3 and C1 could probably be determined by the influence of the shadow of the building adjacent to the school or by different ventilation patterns. The other axis might be related to solar irradiation (and consequently temperature), as microsites (C1, C2, C3, and O), which are potentially influenced by the shadow of the school building, are closely grouped and distant from the others.

**Figure 5:**
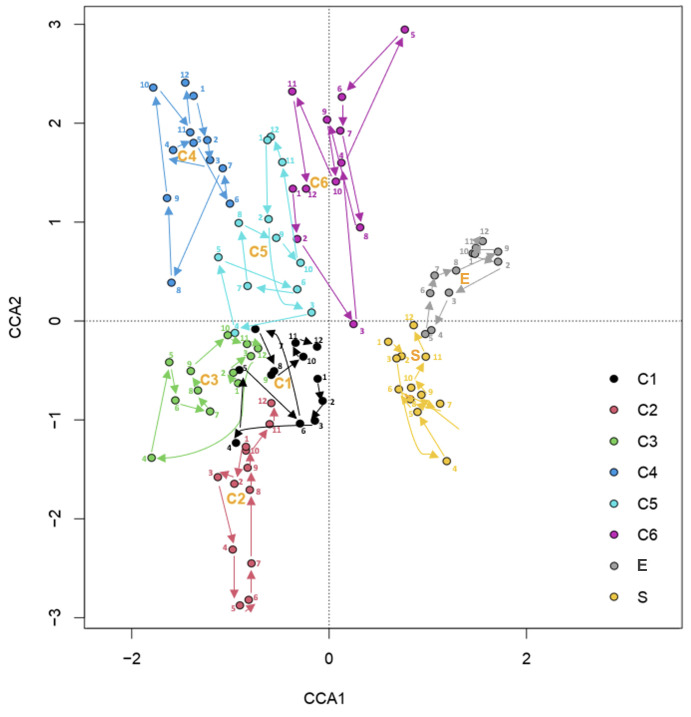
The Canonical-Correlation Analysis (CCA) of the areas on the green roof. The vegetation surveys on the E and S sections of the green roof (in yellow and gray) are well separated from the surveys in the raised garden bed planters (C1–C6), but close to each other. Notes: Colors correspond to the microhabitats of the green roof Figure 7c. Numbers identify the surveys over time (i.e., 1 stands for January, 2 for February, etc.).

We also analyzed the vegetation changes using the Canonical Correlation Analysis (CCA) (Figure 5). This analysis, as shown in Figure 5, highlighted seasonal and monthly trends for each sampling area. The months, numbered from 1 (January) to 12 (December), are marked by the meeting of the tip with the tail of each arrow. It can be seen how, in each sample area, January and December are close, as are the points indicating the summer months. Meanwhile, the points describing Spring and Fall, which typically represent the seasons in which the most significant development of plants occurs, are more spaced out. The different arrows for each cluster almost exactly retrace a complete cycle, mirroring the cycle of a solar year. As highlighted by the PCA and UPGMA, there is a marked separation between the surveys on the E and S sections and the surveys in the raised garden bed planters (C1–C6). The permutation test for the significance of the analysis has a p-value of 0.001. Therefore, the test is significant.

Finally, the results of the IndVal index identified two groups with their most representative species (Table 1). We listed the top five most representative species for each group (group 1: E and S; group 2: C1, C2, C3, C4, C5, and C6). For instance, some species were only collected in the E and S sections of the roof or only in the raised garden bed planters (C1–C6) (with a value of the B index, i.e., specificity, equal to 1, therefore equal to 100%).

## 3. Discussion

The study site is characterized by a Mediterranean climate that poses harsh growing conditions for most plants [40]. During the monitoring period, the site experienced typical Mediterranean climatic conditions, with pronounced summer drought. Specifically, the months from June to August recorded extremely low precipitation, combined with average temperatures generally above 25 °C, creating severe water stress conditions. This was also exacerbated by the fact that the species were located on an unirrigated green roof. These conditions, however, have not affected the growth of several native species that were selected for the initial implementation of the green roof: *Thymus serpyllum, Teucrium chamaedrys, Lavandula stoechas*, and *Saponaria ocymoides* maintained steady growth and cover. These species had already performed well with a regular irrigation regime [60]. These plants are primarily typical of Mediterranean (or South European) clearings, woodland edges, or arid pastures, apart from *Saponaria ocymoides*, which is an orophyte [61]. This last species is adapted to grow on mountain tops, characterized by harsh growing conditions and arid soils with gravel or rocks [61]. Orophytes are indeed adapted to significant daily temperature excursions and intense sunlight, which are similar conditions to those of green roofs. The growth of *Saponaria ocymoides* was also monitored on a green roof in Beijing, where there was no watering; there, the plant showed 100% mortality after a dry period [62]. On the other hand, on a green roof in Berlin, the same plant, without watering, grew well and maintained high cover [63]. Even in our previous study, this species exhibited good flowering and low mortality across all tested irrigation regimes, performing best with higher water inputs [60]. *Thymus serpyllum*, *Teucrium chamaedrys,* and *Lavandula stoechas* have been successfully tested for green roofs in Mediterranean areas, with or without watering (e.g., [49,64]). *Thymus serpillum* demonstrated notable performance in Mediterranean urban conditions, thanks also to its prostrate habit that favors extensive coverage. Other species, such as *Teucrium chamaedrys, Lavandula stoechas,* and *Allium schoenoprasum*, although with different growth habits and therefore different coverage capacities, showed significant resilience, making them promising for use in low-maintenance green roofs. Several successful plants are also melliferous plants that can support insect pollinators (and related ecosystem services).

*Cerastium tomentosum* instead had poor development during our surveys, confirming what we observed in our previous study [60]. This is an endemic species of the central Apennines that thrives on calcareous cliffs with gravel and debris [65], and it is adapted to montane temperate bioclimatic conditions, which are probably too different from those of the green roof of the Keplero High School. Instead, this plant thrived on a green roof in Berlin [63], where the climatic conditions were probably more like those of the natural habitats. The growth of *Cerastium tomentosum* was also monitored on a green roof in southern Italy, but irrigation was supplied as needed [66]. There, *Cerastium tomentosum* appeared to be the best-performing species on average compared to the other tested species [66].

The colonization of green roof habitats is affected by the same factors as in natural habitats [28,29]. These include climatic factors (including moisture availability), abundance and composition of resident plant species (i.e., priority effect and niche overlap) [32], thickness and quality of the substrate (as it can also influence the resident plant cover) [67,68,69], traits of invading species and propagule supply [69,70,71]. The fact that most of our colonists were forbs or grasses may be due to the fact that most of the surviving residents in the raised garden bed planters were Chamaephytes; as such, complementary growth forms could have been favored [29,32]. Conversely, this effect was not observed in the E and S sections of the green roof, where other factors might have had more weight. Another factor that could have influenced the typology of colonizers is the predominant dissemination strategy by wind (Colonization Hypothesis; successful colonizers tend to have tiny seeds and a morphological structure that promotes dispersibility [72]). A wind dispersal dissemination strategy is a functional trait that predominates among plants in urban environments [73,74]. Wind-dispersed plants are also common in arid habitats [72]; therefore, in their dispersion, they might have found on the green roof suitable growing conditions that were not as suitable for other species. Mediterranean plants dispersed by wind were predominant, although the number of exotic species was also relatively high. This could be due to the propagule supply effect as exotic species find their elective habitats in urban contexts. Therophytes might also be predominant for a similar reason, as research suggests that they tend to flourish with high levels of disturbance and nitrogen, typical of urban environments [70]. For the same reason, geophytes tend to decrease and disappear from urban contexts [74].

The difficult climatic and pedological conditions of the green roof have also driven the colonization process and the survival of the colonizers. Therophytes have a short biological cycle and high reproductive capacity, which make them well-adapted to challenging environments and seasonal stressors (i.e., summer drought) [34,69]. Therophytes also tend to be the first colonizers and might disappear when the plant community matures. Mediterranean species may be annual or perennial but are intrinsically adapted to the Mediterranean climate, which is challenging by definition. As such, steno-Mediterranean species might be naturally adapted to the harsh conditions of the green roof. However, the invasion and colonizing process can vary considerably with other temporal and spatial variables [28].

The vegetation analyses showed a predominance of species typical of ruderal communities or rocky environments. Green roofs provide ideal habitats for ruderal plants as they are generally rustic, stress-tolerant, and have high colonization capacity [70,71]. These plants rely on a dynamic process of colonization and regeneration to sustain their populations in the long term [28] (i.e., therophytes rely on this strategy). Ruderal species also predominate during the colonization process examined on other green roofs [29,31,69,70].

Microhabitat heterogeneity, driven by differences in available light, wind exposure, and soil depth, affected the structure, dynamics, and composition of the green roof vegetation. The vegetation analysis identified substantial differences in environmental conditions, with substrate thickness and solar irradiation (and possibly temperature) emerging as the main determining factors in the distribution of plant species. The cluster analysis, PCA, and CCA indicated that certain ecological factors significantly influenced the colonization process. The substrate depth is the most relevant factor, as the vegetation surveys on the E and S sections tend to group despite having different solar exposures. This could be related to the fact that the E and S areas, in contrast to the six raised garden bed planters (C1-C6), present a minimal difference in substrate depth. Deeper soil can retain more moisture [75], give room to more extensive root systems, and thus accommodate a higher number and variety of plant species [69,76] and pedo-fauna [77]. Solar irradiation (and consequently, temperature) is another factor that seems to have some weight, as it can positively affect evapotranspiration and drought stress [29]. Previous studies have analyzed the influence of moisture availability, solar exposure [29,30,78,79], and temperature [80,81], and they have found less plant diversity and lower vegetation cover as moisture decreases and solar exposure increases. Our temporal analysis has not revealed dramatic changes in vegetation composition; however, the CCA has shown a correspondence between plant development and seasonal changes.

Green roofs are novel ecosystems [68,82,83,84] where species occur in new combinations and unusual abundances that differ from those found in natural habitats. On the E and S sections of our green roof, several plants belonged to entirely different vegetation classes (that indicate different ecological preferences and habitats) and only appeared sporadically for a few months (i.e., *Andryala integrifolia*, *Rosa canina*, see Tables S10 and S11). Depending on the seed dispersal and environmental constraints of the green roof, new colonization may be slower than species disappearance (i.e., [85]), and maintaining higher levels of biodiversity may require human interventions [86]. Management could also entail weeding (removing exotics and undesired plants), replacing soil, or adding organic matter [27,85]. A limitation of this study lies in its duration, as plants that initially seem to thrive can later disappear [29,31,87,88,89]. Even so, research has shown that biodiversity may not be sufficient to maximize ecosystem services: species communities on green roofs should also possess diverse functional traits [84,90]. Research on green roofs with native plants shows new directions and opportunities that can offer valid alternatives to *Sedum* mats or low-diversity communities of succulents, especially in Mediterranean areas where studies are still limited.

## 4. Materials and Methods

### 4.1. Climatic Conditions

**Figure 6:**
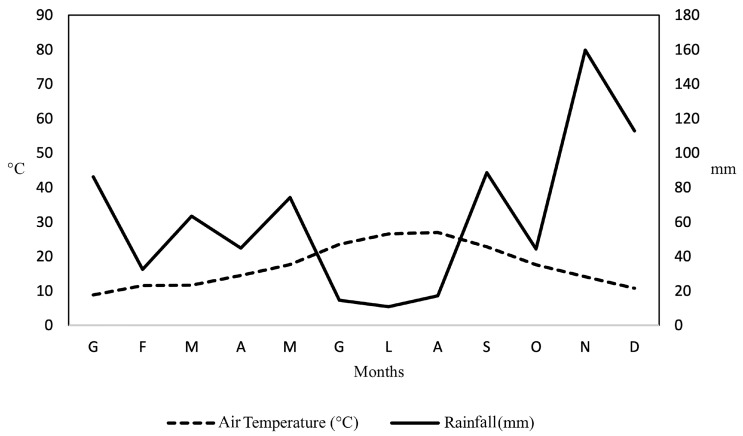
Thermo-pluviometric diagram of the monthly average temperature (°C) and the total amount of rainfall (mm) during the monitoring period (from March 2021 to February 2022). Data were recorded from meteorological instruments in a Stevenson screen near the experimental roof.

The city of Rome, located at an average altitude of 21 m a.s.l. (above sea level), is characterized by a typical Mediterranean climate, which, however, is partially mitigated by the vicinity of the sea [91]. The climate is characterized by mild winters with average minimum temperatures of 4 °C (January–February) and hot summers with average maximum temperatures of 31 °C (July–August). During the study period (2019–2021), the nearest weather station (EUR) recorded monthly temperatures ranging from approximately 10 °C in the winter to 28 °C in the summer. Precipitation showed considerable seasonal variation, with peaks in the Fall (November–December), reaching up to 160 mm, and minimum values during the summer months (June–August) when rainfall dropped below 20 mm (Figure 6).

### 4.2. The Green Roof Structure and Characteristics

**Figure 7:**
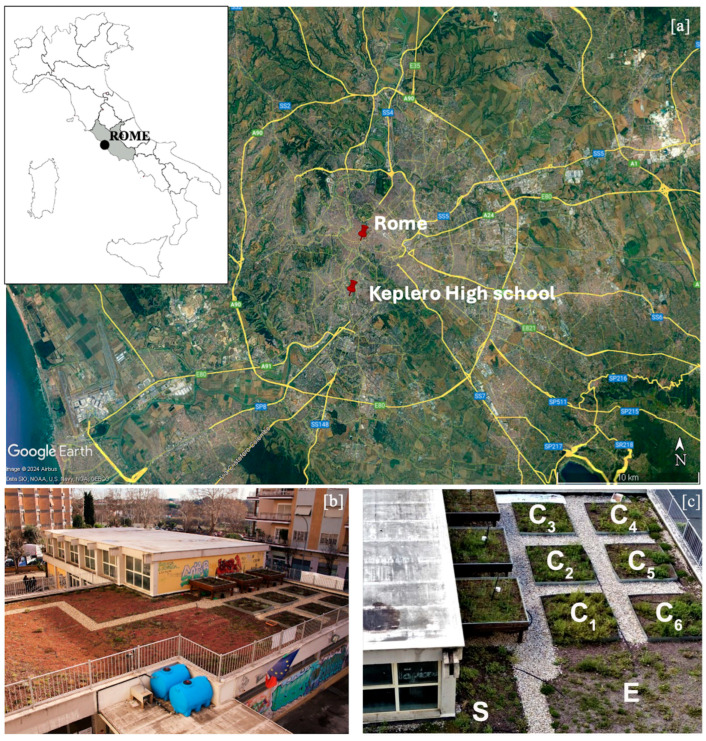
The green roof on top of the Keplero High School. (**a**) Geographical position of the study site; (**b**) Aerial photo of the whole green roof; (**c**) Details of the raised garden bed planters. The more exposed part of the extensive green roof (E) gets sunlight for more extended periods, while the more sheltered part (S) of the extensive green roof is in the shadow for longer; C1–C6 are the raised garden bed planters.

The study was conducted on the Keplero Scientific High School’s green roof in Rome, representing the city’s first green roof installation on a public school building. The site is situated in a densely populated area near the Tiber River (41°86′63″ N, 12°47′24″ E) (Figure 7a). Completed in 2016, the installation comprises a 150 m^2^ extensive green roof, divided into two distinct areas (S-E) by a gravel path and an additional 50 m^2^ experimental section subdivided into six 4 m^2^ test raised garden bed planters (C1–C6), separated from each other by narrow gravel paths and by the raised walls of the planters; the raised planters are contiguous to the rest of the green roof (Figure 7b,c).

The green roof features a multilayered structure incorporating an anti-root waterproofing membrane, a drainage system, a growing substrate, and a vegetation layer. The substrate consists of mineral materials (lapilli, pumice, zeolite) and organic material (peat) with grain sizes ranging from 0.05 to 20 mm and a thickness of 12 cm in the 150 m^2^ area (S-E). The six raised garden bed planters (C1–C6) had a slightly higher thickness (15 cm) (Figure 7c). These areas were considered slightly different for their microclimatic conditions: the S area is in shadow for half the day, whereas the E area is exposed to sunlight for the entire day. The three raised planters, C1, C2, and C3, are in the shade for more extended periods than the C4, C5, and C6 raised planters, thanks to the vicinity of nearby buildings.

The system’s water retention capacity is higher than 85 L/m^2^. According to FLL tests certified by independent institutes, the system’s runoff coefficient (C) is below 0.20. The total hydraulic conductivity of the drainage element at 20 kPa (according to EN ISO 12958 [92]) is not less than 15.00 L/sm at a hydraulic gradient (i) equal to 1 and not less than 1.3 L/sm at i = 0.01. The system is equipped with a drip tube irrigation system, with each tube capable of delivering approximately four liters per hour.

The six raised garden bed planters were used to test the performance of six Mediterranean species (Table 2) that could eventually replace other commonly used species on green roofs through a two-year experiment (2017–2019) [60]. A local floriculture company supplied all the 12 cm potted plants. Six pots of each selected species were planted in each raised garden bed planter in 2016 (October), resulting in a density of nine plants/m^2^ (for a total of 36 plants per raised planter). The remaining green roof area was planted with three *Sedum* species (*S. album* L., *S. reflexum* L., and *S. sexangulare* L.), selected for their compact growth habit (2–5 cm height).

Regular maintenance (removal of wild species) and watering (emergency watering of five minutes per night in the summer months) of the green roof began in March 2017. It continued until the experiment ended in 2019 [60]. Maintenance and irrigation of the green roof completely ceased in the spring of 2020 due to COVID-19 restrictions, leading to changes in vegetation. Despite the lack of watering, some species thrived, and new plants were able to colonize the green roof.

### 4.3. Experimental Design

From March 2021 to February 2022, we carried out 12 monthly vegetation surveys to analyze the behavior of the cultivated species in a no-watering regime and monitor the colonization dynamics of spontaneous vascular plants. During the vegetation surveys, we collated data on the presence and cover of the plants, which varied according to the different stages of the life cycle and development of the species. We divided the green roof surface into eight sampling areas to optimize and better organize the data collection process. The eight sampling areas include the six raised garden bed planters (C1, C2, C3, C4, C5, C6) (4 m^2^ each) and two sub-areas within the rest of the green roof, separated according to irradiance levels: one exposed (E) area (about 85 m^2^) and one sheltered (S) area (about 35 m^2^) (Figure 7c).

We conducted detailed visual assessments within each sampling area to record the presence and cover of both planted and colonizing species. Species were identified using the Flora d’Italia [93], and for each identified species, we indicated life form and chorological type according to standard botanical classifications [93,94]. In order to record cover values, we used the Braun–Blanquet phytosociological method [95]. The Braun–Blanquet methodology entails the identification of plant species within a homogeneous community and evaluating each species’ cover using a simple scale corresponding to percentage cover values. These values range from +, indicating a sporadic presence, to class five (V), indicating a cover estimate between 75% and 100% (Table 3). Even though the Braun–Blanquet method presents some inherent limitations, it is widely used in vegetation studies. In the design phase of the experiment, we decided to use this sampling methodology because the survey areas were well-defined and delimited; within each sample area, we could repeat very accurate surveys every month, counting all individuals, to evaluate even subtle variations in species composition. The methodology’s limitations were accounted for in the interpretation of our results. Each colonizing species was classified according to its phytosociological class of reference [96,97,98]. Ground-level surveys were complemented with drone photography to better visualize broader spatial patterns of vegetation distribution.

### 4.4. Data Analysis

We ran several statistical analyses to evaluate vegetation patterns and community composition. Using the collated life form and chorological type of the species, we calculated biological and chorological spectra, representing their frequency distribution within the plant community, visualized through histograms. Using the matrix of the vegetation surveys, at first, we transformed the Braun–Blanquet [95] cover values into the van der Maarel [99] cover values (Table 3) using the Sørensen index for the quantitative data (Bray–Curtis index) and the PC-Ord software ver. 4.1 [100]. This transformation was necessary to work with a fully numerical scale [99]. We summed the numerical values of each species, then calculated the average to obtain an overall value for each species within each sample area for every month to evaluate their trend and dynamics over time.

Multivariate analyses (cluster analysis, PCA, and CCA) were used to identify differences in environmental conditions among the sections of the green roof. We run a hierarchical cluster analysis of the species matrix using the UPMGA (Unweighted Arithmetic Average Clustering, [101]) method with the chord-transformed data. This method uses the arithmetic mean of the distances (or similarities) between all the elements within a cluster, and the same weight is attributed to each element. We performed a Principal Component Analysis (PCA), which can be used to represent latent (or not immediately apparent) variation within a dataset and visualize the most likely solutions of probabilistic latent variables [102]. We used the PCA method to determine which ecological factors were more likely to drive the plant distribution. We also performed a Canonical Correlation Analysis (CCA), which is used to analyze paired sets of variables [103]. We used the CCA to identify the relationships between clusters and sample areas on the green roof. Finally, we calculated the IndVal index to identify the most representative species of the clusters, i.e., the plants that were specific or more frequent. When the IndVal reaches its maximum value for a species, it means that the species is the most representative of that cluster [104]. We only considered the species with a significant IndVal value (*p* ≤ 0.01). All statistical analyses were performed using the R programming language [105] and PC-Ord version 4.1.

## 5. Conclusions

Our study shows that native Mediterranean species can serve as valid alternatives to *Sedum* carpets, as they are already adapted to the local climate, are preferred by the local fauna, are potentially less expensive, and may require less management work than low-diversity carpets. This preliminary study provided insights into plant survival and colonization dynamics. The spontaneous colonization process can also shed light on potentially interesting plants that can survive and thrive despite the environmental constraints of green roofs. These plants can also enhance the biodiversity of green roofs.

Our research, although with some limitations, still holds potential interest as studies on the colonizing dynamics of green roofs in Mediterranean climates are very limited. Our findings suggest that creating diverse microclimatic conditions can support natural colonization processes, potentially leading to more resilient and biodiverse green roof systems. Ultimately, research on dynamic colonization processes has diverse applications, where developers and designers seek greater biodiversity, a more sustainable approach to long-term management, and possibly enhanced aesthetic appeal.

## Figures and Tables

**Table 1:** The top five most representative species according to the calculation of the IndVal index, with the p-values for each species. The A Index indicates how closely related a species is to a specific grouping in correlation with its frequency. The B index indicates how specific a single species is for the group under analysis. The IndVal index is the arithmetic mean between the A and B indices; thus, it combines specificity with frequency. Only the species with a statistically significant IndVal index (*p* ≤ 0.01) are listed in the table.

**Group 1: (E and S)**	**A**	**B**	**IndVal**	***p*** **. Value**
*Veronica persica*	0.9623	1	0.981	0.001
*Erigeron canadensis*	1	0.9167	0.957	0.001
*Geranium rotundifolium*	0.5917	1	0.769	0.001
*Festuca bromoides*	0.9219	0.5833	0.733	0.005
*Sonchus asper*	0.5124	1	0.716	0.001
**Group 2: C1, C2, C3, C4, C5, C6**	**A**	**B**	**IndVal**	***p*** **. value**
*Helminthotheca echioides*	0.9091	1	0.953	0.001
*Hypochaeris achyrophorus*	0.5732	0.92	0.726	0.001
*Erigeron sumatrensis*	0.5209	1	0.722	0.001
*Medicago sativa*	0.4367	1	0.661	0.009
*Erigeron karviskianus*	0.4008	1	0.633	0.001

**Table 2:** The species that were planted in the six raised garden bed planters on the green roof.

Species	Chorotype	Life Form	Family
*Teucrium chamaedrys* L.	Steno-Medit	Chamaephytes	Lamiaceae
*Lavandula stoechas* L.	Steno-Medit.	Nanophanerophytes	Lamiaceae
*Cerastium tomentosum* L.	Endem. Ital.	Chamaephytes	Caryophyllaceae
*Thymus serpyllum* L.	S-Europ	Chamaephytes	Lamiaceae
*Saponaria ocymoides* L.	Oroph. S-Europ/Oroph. SW-Europ.	Hemicryptophytes	Caryophyllaceae
*Allium schoenoprasum* L.	Eurosiber-Circumbor.	Geophytes	Amaryllidaceae

**Table 3:** Braun–Blanquet cover-abundance scale and van der Maarel’s [99] numerical transformation scale.

Braun–Blanquet Scale	Range of Cover (%)	Van der Maarel’s Ordinal Scale
V	75–100	9
IV	50–75	8
III	25–50	7
II	5–25	5
I	1–5	3
+	<1	2
-	absent	1

## Data Availability

The original contributions presented in this study are included in the article/Supplementary Material. Further inquiries can be directed to the corresponding author.

## Supplementary Materials

The following supporting information can be downloaded at https://www.mdpi.com/article/10.3390/plants14203180/s1: Table S1: Presence and cover of planted species in the sheltered section of the green roof (S); Table S2: Presence and cover of species in the exposed section of the green roof (E); Table S3: Presence and cover of species in the raised garden bed planter 1 (C1); Table S4: Presence and cover of species in the raised garden bed planter 2 (C2); Table S5: Presence and cover of species in the raised garden bed planter 3 (C3); Table S6: Presence and cover of species in the raised garden bed planter 4 (C4); Table S7: Presence and cover of species in the raised garden bed planter 5 (C5); Table S8: Presence and cover of species in the raised garden bed planter 6 (C6); Table S9: List of the species that spontaneously colonized the green roof. They are listed according to their family, from the most to the least represented. Chorotypes and life forms are also provided; Table S10: Vegetation surveys in the sheltered section of the green roof (S); Table S11: Vegetation surveys in the exposed section of the green roof (E); Table S12: Vegetation surveys in the raised garden bed planter 1 (C1); Table S13: Vegetation surveys in the raised garden bed planter 2 (C2); Table S14: Vegetation surveys in the raised garden bed planter 3 (C3); Table S15: Vegetation surveys in the raised garden bed planter 4 (C4); Table S16: Vegetation surveys in the raised garden bed planter 5 (C5); Table S17: Vegetation surveys in the raised garden bed planter 6 (C6).
