# Supplementary material for: How a Green Roof Becomes Biodiverse: Vegetation Analysis on a Green Roof with no Maintenance in Rome (Italy)
Source: Plants (Basel). 2025 Oct 16;14(20):3180. doi: 10.3390/plants14203180 (PMC12567229; doi:10.3390/plants14203180)
Supplement: Supplementary file 1 [file plants-14-03180-s001.zip › plants-3902241-supplementary.pdf]

Supplementary Files

How a green roof becomes biodiverse: Vegetation analysis on a green roof with no maintenance in Rome (Italy)

Bellini A.[1], Savo V.[2*], Caneva G.[1,3], D'Amico E.[1], Casalini R.[4], Bartoli F.[5,1]

[1] Department of Science, Roma Tre University, Viale Marconi 446, 00146 Rome, Italy

[2] Department of Education Science, Roma Tre University, Via del Castro Pretorio 20, 00185 Rome, Italy

[3] National Biodiversity Future Center (NBFC), University of Palermo, Piazza Marina 61, 90133 Palermo, Italy

[4] Liceo Scientifico Statale Giovanni Keplero, Via Silvestro Gherardi, 87, 00146 Rome, Italy

[5] Institute of Heritage Science (CNR-ISPC), National Research Council of Italy, Area della Ricerca di Roma 1, Strada della Neve s.n.c., 00010 Montelibretti (RM), Italy.

Supplementary Table S1. Presence and cover of planted species in the sheltered section of the green roof (S).

Species	Mar 21	Apr 21	May 21	Jun 21	Jul 21	Aug 21	Sep 21	Oct 21	Nov 21	Dec 21	Jan 22	Feb 22
Festuco valesiacae - Brometea erecti												
Allium schoenoprasum	-	-	-	-	-	-	-	-	-	-	-	-
Jacobaea erratica	-	-	-	-	-	-	-	-	-	-	-	-
Saponaria ocymoides	-	-	-	-	-	-	-	-	-	-	-	-
Thymus serpyllum	-	+	1	1	1	1	1	1	1	1	-	-
Thlaspietea rotundifolii												
Cerastium tomentosum	-	-	-	-	-	-	-	-	-	-	-	-
Cisto ladaniferi - Lavanduletea stoechadis												
Lavandula stoechas	-	-	-	-	-	-	-	-	-	-	-	-
Filipendulo ulmariae - Convolvuletea sepium												
Filipendula ulmaria	-	-	-	-	-	-	-	-	-	-	-	-
Trifolio medii - Geranietea sanguinei												
Teucrium chamaedrys	-	-	-	-	-	-	-	-	-	-	-	-

Supplementary Table S2. Presence and cover of species in the exposed section of the green roof (E).

Species	Mar 21	Apr 21	May 21	Jun 21	Jul 21	Aug 21	Sep 21	Oct 21	Nov 21	Dec 21	Jan 22	Feb 22
Festuco valesiacae - Brometea erecti												
Allium schoenoprasum	+	+	-	-	-	-	-	-	1	1	1	+
Jacobaea erratica	+	+	1	1	+	+	+	+	+	+	+	+
Saponaria ocymoides	-	-	-	-	-	-	-	-	-	-	-	-
Thymus serpyllum	+	+	-	-	1	1	1	1	1	1	+	+
Thlaspietea rotundifolii												
Cerastium tomentosum	+	+	-	-	-	-	-	-	-	-	-	+
Cisto ladaniferi - Lavanduletea stoechadis												
Lavandula stoechas	+	+	-	-	-	1	1	1	1	1	1	+
Filipendulo ulmariae - Convolvuletea sepium												
Filipendula ulmaria	+	+	+	-	+	+	+	-	+	+	+	+
Trifolio medii - Geranietea sanguinei												
Teucrium chamaedrys	-	-	-	-	-	-	-	-	-	-	-	-

Supplementary Table S3. Presence and cover of species in the raised garden bed planter 1 (C1).

Species	Mar 21	Apr 21	May 21	Jun 21	Jul 21	Aug 21	Sep 21	Oct 21	Nov 21	Dec 21	Jan 22	Feb 22
Festuco valesiacae - Brometea erecti												
Allium schoenoprasum	-	-	+	+	+	+	1	1	1	1	1	-
Jacobaea erratica	-	-	-	-	-	-	-	-	-	-	-	-
Saponaria ocymoides	+	+	-	-	-	-	-	-	-	+	+	+
Thymus serpyllum	+	2	3	3	3	3	3	2	2	2	+	+
Thlaspietea rotundifolii												
Cerastium tomentosum	-	+	-	-	-	-	-	-	-	-	-	-
Cisto ladaniferi - Lavanduletea stoechadis												
Lavandula stoechas	+	+	+	+	+	+	1	+	+	+	+	+
Filipendulo ulmariae - Convolvuletea sepium												
Filipendula ulmaria	-	-	-	-	-	-	-	-	-	-	-	-
Trifolio medii - Geranietea sanguinei												
Teucrium chamaedrys	1	1	-	-	-	-	-	-	-	1	1	1

Supplementary Table S4. Presence and cover of species in the raised garden bed planter 2 (C2).

**Species**	**Mar 21**	**Apr 21**	**May 21**	**Jun 21**	**Jul 21**	**Aug 21**	**Sep 21**	**Oct 21**	**Nov 21**	**Dec 21**	**Jan 22**	**Feb 22**
Festuco valesiacae - Brometea erecti												
Allium schoenoprasum	-	-	-	-	-	-	-	-	-	-	-	-
Jacobaea erratica	-	-	-	-	-	-	-	-	-	-	-	-
Saponaria ocymoides	+	+	-	-	-	-	-	-	-	-	-	+
Thymus serpyllum	1	2	3	3	3	3	3	3	2	2	1	1
Thlaspietea rotundifolii												
Cerastium tomentosum	-	-	-	-	-	-	-	-	-	-	-	-
Cisto ladaniferi - Lavanduletea stoechadis												
Lavandula stoechas	-	-	-	-	-	-	-	-	-	+	+	+
Filipendulo ulmariae - Convolvuletea sepium												
Filipendula ulmaria	-	-	-	-	-	-	-	-	-	-	-	-
Trifolio medii - Geranietea sanguinei												
Teucrium chamaedrys	-	-	-	-	-	-	-	-	-	-	-	-

Supplementary Table S5. Presence and cover of species in the raised garden bed planter 3 (C3).

**Species**	**Mar 21**	**Apr 21**	**May 21**	**Jun 21**	**Jul 21**	**Aug 21**	**Sep 21**	**Oct 21**	**Nov 21**	**Dec 21**	**Jan 22**	**Feb 22**
Festuco valesiacae - Brometea erecti												
Allium schoenoprasum	-	-	2	2	2	2	2	2	2	2	2	-
Jacobaea erratica	-	-	-	-	-	-	-	-	-	-	-	-
Saponaria ocymoides	+	+	2	2	2	2	2	2	2	1	1	+
Thymus serpyllum	1	2	4	4	4	4	3	3	2	2	1	1
Thlaspietea rotundifolii												
Cerastium tomentosum	-	-	-	-	-	-	-	-	-	-	-	-
Cisto ladaniferi - Lavanduletea stoechadis												
Lavandula stoechas	-	-	-	-	-	-	-	-	-	-	-	-
Filipendulo ulmariae - Convolvuletea sepium												
Filipendula ulmaria	-	-	-	-	-	-	-	-	-	-	-	-
Trifolio medii - Geranietea sanguinei												
Teucrium chamaedrys	1	1	2	2	2	2	2	2	2	2	1	1

Supplementary Table S6. Presence and cover of species in the raised garden bed planter 4 (C4).

**Species**	**Mar 21**	**Apr 21**	**May 21**	**Jun 21**	**Jul 21**	**Aug 21**	**Sep 21**	**Oct 21**	**Nov 21**	**Dec 21**	**Jan 22**	**Feb 22**
Festuco valesiacae - Brometea erecti												
Allium schoenoprasum	-	-	-	-	-	-	-	-	-	-	-	-
Jacobaea erratica	-	-	-	-	-	-	-	-	-	-	-	-
Saponaria ocymoides	1	1	2	2	2	2	2	2	2	1	1	1
Thymus serpyllum	2	1	1	1	1	1	1	1	1	1	2	2
Thlaspietea rotundifolii												
Cerastium tomentosum	-	-	-	-	-	-	-	-	-	-	-	-
Cisto ladaniferi - Lavanduletea stoechadis												
Lavandula stoechas	1	2	2	2	2	2	2	2	2	1	+	+
Filipendulo ulmariae - Convolvuletea sepium												
Filipendula ulmaria	-	-	-	-	-	-	-	-	-	-	-	-
Trifolio medii - Geranietea sanguinei												
Teucrium chamaedrys	1	2	2	2	1	1	1	1	1	+	+	+

Supplementary Table S7. Presence and cover of species in the raised garden bed planters 5 (C5).

Species	Mar 21	Apr 21	May 21	Jun 21	Jul 21	Aug 21	Sep 21	Oct 21	Nov 21	Dec 21	Jan 22	Feb 22
Festuco valesiacae - Brometea erecti												
Allium schoenoprasum	+	+	-	-	-	-	-	-	-	-	-	+
Jacobaea erratica	-	-	-	-	-	-	-	-	-	-	-	1
Saponaria ocymoides	-	-	+	+	+	+	+	+	+	+	+	-
Thymus serpyllum	1	1	2	2	2	2	2	2	2	2	1	1
Thlaspietea rotundifolii												
Cerastium tomentosum	-	-	-	-	-	-	-	-	-	-	-	-
Cisto ladaniferi - Lavanduletea stoechadis												
Lavandula stoechas	-	-	-	-	-	-	-	+	1	1	1	1
Filipendulo ulmariae - Convolvuletea sepium												
Filipendula ulmaria	-	-	-	-	-	-	-	-	-	-	-	-
Trifolio medii - Geranietea sanguinei												
Teucrium chamaedrys	2	2	3	3	3	3	3	3	2	2	2	2

Supplementary Table S8. Presence and cover of species in the raised garden bed planter 6 (C6).

Species	Mar 21	Apr 21	May 21	Jun 21	Jul 21	Aug 21	Sep 21	Oct 21	Nov 21	Dec 21	Jan 22	Feb 22
Festuco valesiacae - Brometea erecti												
Allium schoenoprasum	-	-	-	-	-	-	-	-	-	-	-	-
Jacobaea erratica	-	-	-	-	-	-	-	-	-	-	-	-
Saponaria ocymoides	-	-	+	+	+	+	+	+	+	+	+	-
Thymus serpyllum	1	+	+	+	+	2	1	2	2	2	2	1
Thlaspietea rotundifolii												
Cerastium tomentosum	-	-	-	-	-	-	-	-	-	-	-	-
Cisto ladaniferi - Lavanduletea stoechadis												
Lavandula stoechas	1	2	2	2	2	2	2	2	1	1	1	1
Filipendulo ulmariae - Convolvuletea sepium												
Filipendula ulmaria	-	-	-	-	-	-	-	-	-	-	-	-
Trifolio medii - Geranietea sanguinei												
Teucrium chamaedrys	2	2	-	-	-	-	-	-	-	-	2	2

Supplementary Table S9. List of the species that spontaneously colonized the green roof. They are listed according to their family, from the most to the least represented. Chorotypes and life forms are also provided.

Species	Chorotype	Life form
**Asteraceae**		
Andryala integrifolia L.	Euri-Medit-Occid	T-scap
Crepis bursifolia L.	Medit.	H-scap
Crepis foetida L.	Euri-Medit	H bienn - T scap
Crepis sancta (L.) Bornm.	Medit. Turan	T-scap
Dittrichia viscosa (L.) Greuter	Euri-Medit	H-scap
Erigeron canadensis L.	N-Americ	T-scap
Erigeron karvinskianus DC.	Subtrop. - N-Americ	H-scap
Erigeron sumatrensis Retz.	Americ.	T-scap
Helminthotheca echioides (L.) Holub	Euri-Medit. - Euri - Medit - Orient.	T-scap
Hypochaeris achyrophorus L.	Steno-Medit	T-scap
Hypochaeris glabra L.	Euri-Medit	T-scap

Hypochaeris radicata L.	Europ.-Caucas	H-ros
Lactuca sativa L.	Euri-Medit. - Subsiber.	H scap - H bienn
Picris hieracioides L.	Euroasiat. Eurosiber.	H bienn - H scap
Reichardia picroides (L.) Roth	Steno - Medit	H scap
Senecio vulgaris L.	Cosmop. - EuriMedit - Subcosmop.	T-scap
Sonchus asper (L.) Hill	Euroasiat - Subcosmop.	H bienn - T scap
Sonchus oleraceus L.	Cosmop. Euroasiat. Subcosmop.	H bienn - Tscap
Sonchus tenerrimus L.	Steno-Medit	H scap - T scap
**Poaceae**		
Brachypodium rupestre (Host) Roem. & Schult.	Subatl.	H caesp
Catapodium rigidum (L.) C.E.Hubb.	Euri-Medit	T-scap
Cynodon dactylon (L.) Pers.	Cosmop	Grhiz - Hrept
Echinochloa crus-galli (L.) P. Beauv. subsp. *crus-galli*	Subcomop.	T-scap
Eleusine indica (L.) Gaertn.	Cosmop.	T-scap
Festuca bromoides L.	Paleotemp.	T caesp
Gastridium ventricosum (Gouan) Schinz & Thell.	Medit.Atl.	T-scap
Lolium perenne L.	Circumbor. - Euroasiat.	H caesp
Poa annua L.	Cosmop.	T caesp
Setaria pumila (Poir.) Roem. & Schult.	Subcosmp	T scap
**Fabaceae**		
Lathyrus tuberosus L.	Paleotemp.	H scap
Lotus ornithopodioides L.	Steno-Medit.	T scap
Medicago minima (L.) L.	Euri-Medit.	T scap
Medicago polymorpha L.	Euri - Medit. Subcosmop.	T scap
Medicago sativa L.	Eurasiat. - Steno - Medit	H-scap
Trigonella italica (L.) Coulot & Rabaute	N-Medit	T-scap
**Lamiaceae**		
Clinopodium nepeta (L.) Kuntze subsp. *nepeta*	Medit- Mont. - Steno - Medit	Ch suffr/ H scap
Lamium amplexicaule L.	Euroasiat - Paleotemp	T scap
Mentha spicata L.	Euri-Medit	H-scap
Micromeria graeca (L.) Benth. ex Rchb.	Steno-Medit	Ch suffr
**Euphorbiaceae**		
Euphorbia maculata L.	N-Americ.	T-rept
Euphorbia prostrata Aiton	N-Americ	T-rept
Mercurialis annua L.	Paleotemp	T scap
**Plantaginaceae**		
Cymbalaria muralis G.Gaertn., B.Mey. & Scherb.	S-Europ + Subcosmop.	Ch rept - H scap
Platanus hispanica Mill. ex Münchh.	Euri - Medit.	P scap
Veronica persica Poir.	Centroeurop. - SE.Europ.	T scap
**Rosaceae**		
Potentilla reptans L.	Paleotemp. - Subcosmop	H ros
Rosa canina L.	Paleotemp.	NP

Rubus ulmifolius Schott	Europ. - Euri-Medit	NP - P caesp
**Caryophyllaceae**		
Fallopia convolvulus (L.) Á.Löve	Circumbor.	T-scap
Stellaria media (L.) Vill.	Cosmop.	H bienn - T rept
**Papaveraceae**		
Fumaria capreolata L. subsp. *capreolata*	Euri-medit. Steno-medit	T-scap
Fumaria officinalis L.	Euroasiat - Paleotemp - Subcosmop.	T-scap
**Apiaceae**		
Orlaya grandiflora (L.) Hoffm.	Centroeurop. - Pontica	T-scap
**Campanulaceae**		
Campanula erinus L.	Steno-Medit	T-scap
**Digitarieae**		
Digitaria sanguinalis (L.) Scop.	Cosmop.	T scap
**Gentiaceae**		
Blackstonia perfoliata (L.) Huds.	Euri-Medit	T-scap
**Geraniaceae**		
Geranium rotundifolium L.	Paleotemp. - Subcosmop	T-scap
**Oxalidaceae**		
Oxalis corniculata L.	Cosmop. - EuriMedit - Subcosmop.	Ch rept - H reptt
**Portulacaceae**		
Portulaca oleracea L.	Subcosmop	T scap
**Ranunculaceae**		
Nigella damascena L.	Euri-Medit, Steno-Medit	T-scap
**Saxifragaceae**		
Saxifraga adscendens L.	Orof. Centroeurop - Orof. S-Europ	H bienn
**Solanaceae**		
Solanum nigrum L.	Cosmop. Eurasiat.	T scap

Supplementary Table S10. Vegetation surveys in the sheltered section of the green roof (O).

Species	Mar 21	Apr 21	May 21	Jun 21	Jul 21	Aug 21	Sep 21	Oct 21	Nov 21	Dec 21	Jan 22	Feb 22
Stellarienea mediae												
Andryala integrifolia L.	-	-	+	-	-	-	-	-	-	-	-	-
Digitaria sanguinalis (L.) Scop.	-	-	-	-	-	-	-	-	-	1	+	+
Fallopia convolvulus (L.) Á.Löve	-	-	-	-	-	-	-	-	-	-	-	-
Fumaria officinalis L.	+	+	-	-	-	-	-	-	-	-	-	+
Helminthotheca echioides (L.) Holub	-	-	-	-	-	-	-	-	-	-	-	-
Lathyrus tuberosus L.	-	-	-	-	-	-	-	-	-	-	-	-
Mercurialis annua L.	-	-	-	-	-	-	-	-	-	-	+	+
Nigella damascena L.	-	-	+	+	-	-	-	-	-	-	-	-
Orlaya grandiflora (L.) Hoffm.	-	-	-	-	-	-	-	-	-	-	-	-
Setaria pumila (Poir.) Roem. & Schult.	-	-	-	-	-	-	-	-	-	-	-	-
Sonchus asper (L.) Hill	+	1	1	1	1	+	+	+	+	+	+	+
Sonchus oleraceus L.	+	3	2	1	1	+	+	+	+	+	+	+
Veronica persica Poir.	+	1	+	+	+	+	+	+	+	+	+	+
Chenopodio - stellarienea												
Crepis bursifolia L.	-	+	+	+	+	-	-	-	+	+	-	+
Crepis foetida L.	-	-	-	+	+	-	-	-	-	-	-	-
Crepis sancta (L.) Bornm.	-	1	+	+	+	-	-	+	+	+	-	+
Erigeron canadensis L.	2	1	+	+	+	+	+	+	+	+	-	-
Erigeron sumatrensis Retz.	+	3	2	1	1	1	1	1	+	+	-	-
Fumaria capreolata L. subsp. *capreolata*	-	-	-	-	-	-	-	-	-	-	-	-
Gastridium ventricosum (Gouan) Schinz & Thell.	-	-	-	-	-	-	-	-	-	-	-	-
Lactuca sativa L.	-	-	-	-	-	-	-	-	-	-	-	+
Medicago polymorpha L.	-	-	-	-	-	-	-	-	+	+	+	+
Senecio vulgaris L.	-	-	-	-	-	-	+	+	+	+	+	+
Solanum nigrum L.	+	+	+	+	+	+	+	+	-	+	+	+
Stellaria media (L.) Vill.	-	-	-	-	-	-	-	-	-	-	-	+
Trigonella italica (L.) Coulot & Rabaute	+	+	-	-	-	-	-	-	-	-	-	-
Parietarietea judaicae												
Cymbalaria muralis G.Gaertn., B.Mey. & Scherb.	-	-	-	-	-	-	-	-	-	-	-	-
Erigeron karvinskianus DC.	-	1	2	1	1	+	+	+	+	+	-	-
Sonchus tenerrimus L.	2	1	2	+	+	+	+	+	+	+	+	+
Reichardia picroides (L.) Roth	-	-	-	-	-	-	-	-	-	-	-	-
Polygono arenastri - Poetea annuae												
Euphorbia maculata L.	-	-	-	+	+	+	2	+	1	1	-	-
Euphorbia prostrata Aiton	-	-	-	-	-	-	-	-	-	+	+	-
Oxalis corniculata L.	3	+	+	+	+	+	+	-	-	-	-	-
Poa annua L.	-	-	-	-	-	-	-	-	-	-	-	-
Portulaca oleracea L.	-	-	-	-	-	-	-	-	-	-	-	-
Tuberarietea guttate												
Eleusine indica (L.) Gaertn.	-	-	-	-	-	-	-	+	+	+	-	-
Hypochaeris achyrophorus L.	-	-	-	-	-	-	-	-	-	-	-	+
Lotus ornithopodioides L.	-	-	-	-	-	-	-	-	-	-	-	-
Agrostietea stoloniferae												
Mentha spicata L.	-	-	-	-	-	-	-	-	-	-	-	-
Potentilla reptans L.	-	-	-	-	-	-	-	-	-	-	-	-
Molinio - Arrhenatheretea												
Cynodon dactylon (L.) Pers.	1	1	1	1	1	1	1	1	1	-	-	-
Lolium perenne L.	-	-	-	-	-	-	-	-	-	-	-	-
Saginetea maritimae												
Catapodium rigidum (L.) C.E.Hubb.	-	-	-	-	-	-	-	-	-	-	-	-
Hypochaeris glabra L.	-	-	-	-	-	-	-	-	-	-	-	-
Thlaspietea rotundifolii												
Dittrichia viscosa (L.) Greuter	-	-	-	-	-	-	-	-	-	-	-	-
Micromeria graeca (L.) Benth. ex Rchb.	-	-	-	+	+	+	+	+	+	+	-	-
Trifolio medii - Geranietea sanguinei												
Brachypodium rupestre (Host) Roem. & Schult.	-	-	-	-	-	-	-	-	-	-	-	-
Clinopodium nepeta (L.) Kuntze subsp. *nepeta*	-	1	1	1	1	+	+	+	1	1	+	+
Anomodonto - Polypodietea cambrici												
Saxifraga adscendens L.	-	-	-	-	-	-	-	-	-	-	-	-
Asplenietea trichomanis												
Lamium amplexicaule L.	-	-	-	-	-	-	-	-	-	-	-	-
Bidentetea tripartitae												
Echinochloa crus-galli (L.) P.Beauv. subsp. crus-galli	-	-	-	-	-	-	-	-	-	-	-	-
Cardaminetea hirsutae												
Campanula erinus L.	+	+	+	-	-	-	-	-	-	-	-	-
Galio aparines - urticetea dioicae												
Bituminaria bituminosa (L.) C.H.Stirt.	-	-	-	-	-	-	-	-	-	-	-	-
Isoeto-nano juncetae												
Blackstonia perfoliata (L.) Huds.	2	2	2	2	1	+	-	-	-	-	-	-
Koelerio glaucae - Corynephoretea canescentis												
Hypochaeris radicata L.	-	-	-	-	-	-	-	-	-	-	-	-
Rhamno catharticae - Prunetea spinosae												
Rosa canina L.	-	-	-	-	-	-	-	-	-	-	-	-
Rubus ulmifolius Schott	-	-	-	-	-	-	-	-	-	-	-	-
Salici purpureae - Populetea nigrae												
Platanus hispanica Mill. ex Münchh.	-	-	-	-	-	-	-	-	-	-	-	-
Sedo albi - Sclerentetea biennis												
Medicago minima (L.) L.	-	1	1	-	-	-	-	-	-	-	-	-

Supplementary Table S11. Vegetation surveys in the exposed section of the green roof (S).

Species	Mar 21	Apr 21	May 21	Jun 21	Jul 21	Aug 21	Sep 21	Oct 21	Nov 21	Dec 21	Jan 22	Feb 22
Stellarienea mediae												
Andryala integrifolia L.	-	-	-	-	-	-	-	-	-	-	-	-
Digitaria sanguinalis (L.) Scop.	-	-	-	-	-	-	-	-	-	+	+	+
Fallopia convolvulus (L.) Á.Löve	-	-	-	-	-	-	-	-	-	-	-	-
Fumaria officinalis L.	+	+	-	-	-	-	-	-	+	+	+	+
Helminthotheca echioides (L.) Holub	-	-	-	-	-	-	-	-	-	-	-	-
Lathyrus tuberosus L.	-	-	-	-	-	-	-	-	-	-	-	-
Mercurialis annua L.	-	-	-	-	-	-	-	-	-	-	-	-
Nigella damascena L.	-	-	1	1	+	+	-	-	-	-	-	-
Orlaya grandiflora (L.) Hoffm.	-	-	+	+	+	+	-	-	-	-	-	-
Setaria pumila (Poir.) Roem. & Schult.	-	-	-	-	+	+	+	+	+	+	+	+
Sonchus asper (L.) Hill	2	2	1	1	+	+	+	+	+	1	+	+
Sonchus oleraceus L.	2	2	1	1	+	+	+	+	+	1	1	+
Veronica persica Poir.	+	+	+	+	+	+	+	+	+	+	+	+
Chenopodio - stellarienea												
Crepis bursifolia L.	+	+	+	+	+	-	-	-	1	1	1	1
Crepis foetida L.	-	-	-	+	-	-	-	-	-	-	-	-
Crepis sancta (L.) Bornm.	-	+	+	+	+	-	-	-	+	+	+	+
Erigeron canadensis L.	3	3	+	+	+	+	+	+	+	+	+	+
Erigeron sumatrensis Retz.	+	2	2	2	2	1	1	1	1	+	+	+
Fumaria capreolata L. subsp. *capreolata*	-	-	-	-	-	-	-	-	+	+	+	-
Gastridium ventricosum (Gouan) Schinz & Thell.	-	-	+	+	-	-	-	-	-	-	-	-
Lactuca sativa L.	-	-	-	-	-	-	-	-	-	-	-	+
Medicago polymorpha L.	-	-	+	+	+	+	+	+	+	+	+	+
Senecio vulgaris L.	-	-	-	-	-	-	-	-	-	-	+	+
Solanum nigrum L.	-	-	-	-	-	-	-	-	+	1	+	+
Stellaria media (L.) Vill.	-	-	-	-	-	-	-	-	-	-	+	+
Trigonella italica (L.) Coulot & Rabaute	2	2	2	2	+	+	+	+	+	+	+	+
Parietarietea judaicae												
Cymbalaria muralis G.Gaertn., B.Mey. & Scherb.	-	-	-	-	-	-	-	-	-	-	-	-
Erigeron karvinskianus DC.	-	2	2	2	2	+	1	1	1	+	+	+
Sonchus tenerrimus L.	3	2	1	1	+	+	+	+	+	+	+	+
Reichardia picroides (L.) Roth	-	-	-	-	-	-	-	-	-	-	-	-
Polygono arenastri - Poetea annuae												
Euphorbia maculata L.	-	-	-	-	2	1	1	1	1	1	1	-
Euphorbia prostrata Aiton	-	-	-	1	-	-	-	-	-	-	-	-
Oxalis corniculata L.	-	-	-	-	-	-	-	-	-	+	+	+
Poa annua L.	-	-	-	-	-	-	-	-	-	-	-	-
Portulaca oleracea L.	-	-	-	-	-	+	+	+	-	-	+	-
Tuberarietea guttate												
Eleusine indica (L.) Gaertn.	-	-	-	-	-	-	-	-	+	+	+	+
Hypochaeris achyrophorus L.	+	-	-	-	-	-	-	-	+	+	-	+
Lotus ornithopodioides L.	-	-	-	-	-	-	-	-	-	-	-	-
Agrostietea stoloniferae												
Mentha spicata L.	-	-	-	+	1	+	+	+	+	+	+	+
Potentilla reptans L.	-	-	-	-	-	-	-	-	-	-	-	-
Molinio - Arrhenatheretea												
Cynodon dactylon (L.) Pers.	+	+	+	+	+	+	+	+	+	+	+	+
Lolium perenne L.	-	-	-	-	-	-	-	-	-	-	-	-
Saginetea maritimae												
Catapodium rigidum (L.) C.E.Hubb.	-	-	-	-	-	-	-	-	-	-	-	-
Hypochaeris glabra L.	-	-	-	-	-	-	-	-	-	-	-	-
Thlaspietea rotundifolii												
Dittrichia viscosa (L.) Greuter	-	-	-	-	-	-	-	-	-	-	-	-
Micromeria graeca (L.) Benth. ex Rchb.	-	-	-	+	1	+	+	+	+	+	-	-
Trifolio medii - Geranietea sanguinei												
Brachypodium rupestre (Host) Roem. & Schult.	-	-	-	-	+	+	+	+	+	+	+	-
Clinopodium nepeta (L.) Kuntze subsp. *nepeta*	4	3	2	+	+	+	+	+	1	1	+	+
Anomodonto - Polypodietea cambrici												
Saxifraga adscendens L.	-	-	-	-	-	-	-	-	-	-	-	-
Asplenietea trichomanis												
Lamium amplexicaule L.	-	-	-	-	-	-	-	-	+	+	+	-
Bidentetea tripartitae												
Echinochloa crus-galli (L.) P.Beauv. subsp. crus-galli	-	-	-	-	+	+	+	+	+	+	+	+
Cardaminetea hirsutae												
Campanula erinus L.	-	-	-	-	-	-	-	-	-	-	-	-
Galio aparines - urticetea dioicae												
Bituminaria bituminosa (L.) C.H.Stirt.	-	-	-	-	-	-	-	-	+	+	+	+
Isoeto-nano juncetae												
Blackstonia perfoliata (L.) Huds.	1	1	1	+	+	+	-	-	-	-	-	-
Koelerio glaucae - Corynephoretea canescentis												
Hypochaeris radicata L.	-	-	-	-	-	-	-	-	-	-	-	-
Rhamno catharticae - Prunetea spinosae												
Rosa canina L.	+	+	+	-	-	-	-	-	-	-	-	-
Rubus ulmifolius Schott	-	-	-	-	-	-	-	-	-	-	-	-
Salici purpureae - Populetea nigrae												
Platanus hispanica Mill. ex Münchh.	-	-	-	-	-	-	-	-	-	-	-	-
Sedo albi - Sclerentetea biennis												
Medicago minima (L.) L.	-	1	1	1	+	+	+	+	+	+	-	-

Supplementary Table S12. Vegetation surveys in the raised garden bed planter 1 (C1).

Species	Mar 21	Apr 21	May 21	Jun 21	Jul 21	Aug 21	Sep 21	Oct 21	Nov 21	Dec 21	Jan 22	Feb 22
Stellarienea mediae												
Andryala integrifolia L.	-	-	-	-	-	-	-	-	-	-	-	-
Digitaria sanguinalis (L.) Scop.	-	-	-	-	-	-	-	-	-	+	+	+
Fallopia convolvulus (L.) Á.Löve	-	-	-	-	-	-	-	-	-	-	-	-
Fumaria officinalis L.	-	-	-	-	-	-	-	-	-	-	-	-
Helminthotheca echioides (L.) Holub	-	-	-	-	-	-	-	-	-	-	-	-
Lathyrus tuberosus L.	-	-	-	-	-	-	-	-	-	-	-	-
Mercurialis annua L.	-	-	-	-	-	-	-	-	-	-	-	-
Nigella damascena L.	-	-	-	-	-	-	-	-	-	-	-	-
Orlaya grandiflora (L.) Hoffm.	-	-	-	-	-	-	-	-	-	-	-	-
Setaria pumila (Poir.) Roem. & Schult.	-	-	-	-	-	-	-	-	-	-	-	-
Sonchus asper (L.) Hill	-	-	+	+	+	+	+	-	-	1	2	2
Sonchus oleraceus L.	-	-	1	1	1	+	+	+	1	1	2	2
Veronica persica Poir.	-	-	-	-	-	-	-	-	-	-	-	-
Chenopodio - stellarienea												
Crepis bursifolia L.	-	-	-	-	-	-	-	+	+	+	+	+
Crepis foetida L.	-	-	-	-	-	-	-	-	-	-	-	-
Crepis sancta (L.) Bornm.	-	-	-	-	-	-	-	-	-	-	-	-
Erigeron canadensis L.	-	-	-	-	-	-	-	-	-	-	-	-
Erigeron sumatrensis Retz.	-	2	2	3	3	3	3	3	+	+	+	+
Fumaria capreolata L. subsp. *capreolata*	-	-	-	-	-	-	-	-	-	-	-	-
Gastridium ventricosum (Gouan) Schinz & Thell.	-	-	-	-	-	-	-	-	-	-	-	-
Lactuca sativa L.	+	+	-	1	1	+	+	+	-	-	+	1
Medicago polymorpha L.	-	-	-	-	-	-	-	-	-	-	-	-
Senecio vulgaris L.	-	-	-	-	-	-	-	-	-	+	+	+
Solanum nigrum L.	+	+	+	+	+	+	+	+	+	+	+	+
Stellaria media (L.) Vill.	-	-	-	-	-	-	-	-	-	-	-	-
Trigonella italica (L.) Coulot & Rabaute	4	5	4	4	-	+	+	1	1	2	+	+
Parietarietea judaicae												
Cymbalaria muralis G.Gaertn., B.Mey. & Scherb.	-	-	-	-	-	-	-	-	-	-	-	-
Erigeron karvinskianus DC.	+	+	+	+	+	+	+	+	+	+	1	1
Sonchus tenerrimus L.	+	+	+	+	+	+	+	+	1	1	2	2
Reichardia picroides (L.) Roth	+	+	+	+	+	+	+	-	-	-	-	-
Polygono arenastri - Poetea annuae												
Euphorbia maculata L.	-	-	-	-	2	1	2	2	2	2	2	-
Euphorbia prostrata Aiton	-	-	-	2	-	-	-	-	-	-	-	-
Oxalis corniculata L.	-	-	+	-	-	-	-	-	-	+	+	+
Poa annua L.	-	-	-	-	-	-	-	-	-	-	-	-
Portulaca oleracea L.	-	-	-	-	-	-	+	+	-	-	-	-
Tuberarietea guttate												
Eleusine indica (L.) Gaertn.	-	-	-	-	-	-	-	-	-	-	-	-
Hypochaeris achyrophorus L.	-	-	-	-	-	-	-	+	+	+	+	+
Lotus ornithopodioides L.	-	-	-	-	-	-	-	-	-	-	-	-
Agrostietea stoloniferae												
Mentha spicata L.	-	-	-	-	-	-	-	-	-	-	-	-
Potentilla reptans L.	-	-	-	-	-	-	-	-	-	-	-	-
Molinio - Arrhenatheretea												
Cynodon dactylon (L.) Pers.	-	-	-	-	-	-	+	+	+	-	-	-
Lolium perenne L.	-	-	-	-	-	-	-	-	-	-	-	-
Saginetea maritimae												
Catapodium rigidum (L.) C.E.Hubb.	-	-	-	-	-	-	-	-	-	-	-	-
Hypochaeris glabra L.	-	-	-	-	-	-	-	-	-	-	-	-
Thlaspietea rotundifolii												
Dittrichia viscosa (L.) Greuter	-	-	-	-	-	-	-	-	-	-	-	-
Micromeria graeca (L.) Benth. ex Rchb.	-	-	-	+	+	+	+	+	+	+	+	+
Trifolio medii - Geranietea sanguinei												
Brachypodium rupestre (Host) Roem. & Schult.	-	-	-	-	-	-	-	-	-	-	-	-
Clinopodium nepeta (L.) Kuntze subsp. *nepeta*	-	-	+	+	+	+	+	+	+	+	+	+
Anomodonto - Polypodietea cambrici												
Saxifraga adscendens L.	-	-	-	-	-	-	-	-	-	-	-	-
Asplenietea trichomanis												
Lamium amplexicaule L.	-	-	-	-	-	-	-	-	-	-	-	-
Bidentetea tripartitae												
Echinochloa crus-galli (L.) P.Beauv. subsp. crus-galli	-	-	-	-	-	-	-	-	-	-	-	-
Cardaminetea hirsutae												
Campanula erinus L.	-	-	-	-	-	-	-	-	-	-	-	-
Galio aparines - urticetea dioicae												
Bituminaria bituminosa (L.) C.H.Stirt.	-	-	-	-	-	-	-	-	-	-	-	-
Isoeto-nano juncetae												
Blackstonia perfoliata (L.) Huds.	-	-	-	-	-	-	-	-	-	-	-	-
Koelerio glaucae - Corynephoretea canescentis												
Hypochaeris radicata L.	-	-	-	-	-	-	-	-	-	-	-	-
Rhamno catharticae - Prunetea spinosae												
Rosa canina L.	-	-	-	-	-	-	-	-	-	-	-	-
Rubus ulmifolius Schott	-	-	-	-	-	-	-	-	-	-	-	-
Salici purpureae - Populetea nigrae												
Platanus hispanica Mill. ex Münchh.	-	-	-	-	-	-	-	-	-	-	-	-
Sedo albi - Sclerentetea biennis												
Medicago minima (L.) L.	-	-	-	-	-	-	-	-	-	-	-	-

Supplementary Table S13. Vegetation surveys in the raised garden bed planter 2 (C2).

Species	Mar 21	Apr 21	May 21	Jun 21	Jul 21	Aug 21	Sep 21	Oct 21	Nov 21	Dec 21	Jan 22	Feb 22
Stellarienea mediae												
Andryala integrifolia L.	-	-	-	-	-	-	-	-	-	-	-	-
Digitaria sanguinalis (L.) Scop.	-	-	-	-	-	-	-	-	-	+	+	+
Fallopia convolvulus (L.) Á.Löve	-	-	-	-	-	-	-	-	-	-	-	-
Fumaria officinalis L.	-	-	-	-	-	-	-	-	-	-	-	-
Helminthotheca echioides (L.) Holub	+	-	-	-	-	-	-	-	-	-	-	-
Lathyrus tuberosus L.	-	-	-	-	-	-	-	-	-	-	-	-
Mercurialis annua L.	-	-	-	-	-	-	-	-	-	-	-	-
Nigella damascena L.	-	-	-	-	-	-	-	-	-	-	-	-
Orlaya grandiflora (L.) Hoffm.	-	-	-	-	-	-	-	-	-	-	-	-
Setaria pumila (Poir.) Roem. & Schult.	-	-	-	-	-	-	-	-	-	-	-	-
Sonchus asper (L.) Hill	-	-	-	-	-	-	-	-	1	1	2	1
Sonchus oleraceus L.	-	-	-	-	-	-	-	+	1	1	2	2
Veronica persica Poir.	-	-	-	-	-	-	-	-	-	-	-	-
Chenopodio - stellarienea												
Crepis bursifolia L.	-	+	+	+	+	+	+	1	1	1	2	1
Crepis foetida L.	-	-	-	-	-	-	-	-	-	-	-	-
Crepis sancta (L.) Bornm.	-	-	-	-	-	-	-	-	-	-	-	-
Erigeron canadensis L.	-	-	-	-	-	-	-	-	-	-	-	-
Erigeron sumatrensis Retz.	-	2	4	3	3	3	3	3	+	+	+	+
Fumaria capreolata L. subsp. *capreolata*	-	-	-	-	-	-	-	-	-	-	-	-
Gastridium ventricosum (Gouan) Schinz & Thell.	-	-	-	-	-	-	-	-	-	-	-	-
Lactuca sativa L.	-	-	-	-	-	-	-	-	-	-	+	1
Medicago polymorpha L.	-	-	-	-	-	-	-	-	-	-	-	-
Senecio vulgaris L.	-	-	-	-	-	-	-	-	-	-	-	-
Solanum nigrum L.	-	1	-	-	-	-	-	-	-	-	-	-
Stellaria media (L.) Vill.	-	-	-	-	-	-	-	-	-	-	-	-
Trigonella italica (L.) Coulot & Rabaute	+	1	1	1	-	-	-	+	1	1	+	+
Parietarietea judaicae												
Cymbalaria muralis G.Gaertn., B.Mey. & Scherb.	-	-	-	-	-	-	-	-	-	-	-	-
Erigeron karvinskianus DC.	4	3	3	2	2	2	2	2	2	2	2	1
Sonchus tenerrimus L.	-	-	-	-	-	-	-	+	1	1	2	2
Reichardia picroides (L.) Roth	-	-	-	-	-	-	-	-	-	-	-	-
Polygono arenastri - Poetea annuae												
Euphorbia maculata L.	-	-	-	-	-	-	-	-	-	-	-	-
Euphorbia prostrata Aiton	-	-	-	-	-	-	-	-	-	-	-	-
Oxalis corniculata L.	-	-	-	-	-	-	-	-	-	-	-	-
Poa annua L.	-	-	-	-	-	-	-	-	-	-	-	+
Portulaca oleracea L.	-	-	-	-	-	-	-	-	-	-	-	-
Tuberarietea guttate												
Eleusine indica (L.) Gaertn.	-	-	-	-	-	-	-	-	-	-	-	-
Hypochaeris achyrophorus L.	-	-	-	-	-	-	-	-	2	2	2	1
Lotus ornithopodioides L.	-	+	1	1	-	-	-	-	-	-	+	+
Agrostietea stoloniferae												
Mentha spicata L.	-	-	-	-	-	-	-	-	-	-	-	-
Potentilla reptans L.	-	-	-	-	-	-	-	-	-	-	-	-
Molinio - Arrhenatheretea												
Cynodon dactylon (L.) Pers.	-	-	-	-	-	-	+	+	+	-	-	-
Lolium perenne L.	-	-	-	-	-	-	-	-	-	-	-	-
Saginetea maritimae												
Catapodium rigidum (L.) C.E.Hubb.	-	-	-	-	-	-	-	-	-	-	-	-
Hypochaeris glabra L.	-	+	-	-	-	-	-	-	-	-	-	-
Thlaspietea rotundifolii												
Dittrichia viscosa (L.) Greuter	-	-	-	-	-	-	-	-	-	+	+	+
Micromeria graeca (L.) Benth. ex Rchb.	-	-	-	+	+	+	+	+	+	+	-	-
Trifolio medii - Geranietea sanguinei												
Brachypodium rupestre (Host) Roem. & Schult.	-	-	-	-	-	-	-	-	-	-	-	-
Clinopodium nepeta (L.) Kuntze subsp. *nepeta*	-	-	-	-	-	-	-	-	-	-	-	-
Anomodonto - Polypodietea cambrici												
Saxifraga adscendens L.	-	-	-	-	-	-	-	-	-	-	-	-
Asplenietea trichomanis												
Lamium amplexicaule L.	-	-	-	-	-	-	-	-	-	-	-	-
Bidentetea tripartitae												
Echinochloa crus-galli (L.) P.Beauv. subsp. crus-galli	-	-	-	-	-	-	-	-	-	-	-	-
Cardaminetea hirsutae												
Campanula erinus L.	-	-	-	-	-	-	-	-	-	-	-	-
Galio aparines - urticetea dioicae												
Bituminaria bituminosa (L.) C.H.Stirt.	-	-	-	-	-	-	-	-	-	-	-	-
Isoeto-nano juncetae												
Blackstonia perfoliata (L.) Huds.	+	+	1	1	+	+	-	-	-	-	-	-
Koelerio glaucae - Corynephoretea canescentis												
Hypochaeris radicata L.	-	-	-	-	-	-	-	-	-	-	-	-
Rhamno catharticae - Prunetea spinosae												
Rosa canina L.	-	-	-	-	-	-	-	-	-	-	-	-
Rubus ulmifolius Schott	-	-	-	-	-	-	-	-	-	-	-	-
Salici purpureae - Populetea nigrae												
Platanus hispanica Mill. ex Münchh.	-	-	-	-	-	-	-	-	-	-	-	-
Sedo albi - Sclerentetea biennis												
Medicago minima (L.) L.	-	-	-	-	-	-	-	-	-	-	-	-

Supplementary Table S14. Vegetation surveys in the raised garden bed planter 3 (C3).

Species	Mar 21	Apr 21	May 21	Jun 21	Jul 21	Aug 21	Sep 21	Oct 21	Nov 21	Dec 21	Jan 22	Feb 22
Stellarienea mediae												
Andryala integrifolia L.	-	-	-	-	-	-	-	-	-	-	-	-
Digitaria sanguinalis (L.) Scop.	-	-	-	-	-	-	-	-	-	-	+	+
Fallopia convolvulus (L.) Á.Löve	-	-	-	-	-	-	-	-	-	-	-	-
Fumaria officinalis L.	-	-	-	-	-	-	-	-	-	-	-	-
Helminthotheca echioides (L.) Holub	+	+	+	+	-	-	-	-	-	-	-	-
Lathyrus tuberosus L.	-	-	-	-	-	-	-	-	-	-	-	-
Mercurialis annua L.	-	-	-	-	-	-	-	-	-	-	-	-
Nigella damascena L.	-	-	-	-	-	-	-	-	-	-	-	-
Orlaya grandiflora (L.) Hoffm.	-	-	-	-	-	-	-	-	-	-	-	-
Setaria pumila (Poir.) Roem. & Schult.	-	-	-	-	-	-	-	-	-	-	-	-
Sonchus asper (L.) Hill	-	-	+	+	-	-	-	-	+	+	+	+
Sonchus oleraceus L.	-	-	-	-	-	-	-	-	+	+	+	1
Veronica persica Poir.	-	-	-	-	-	-	-	-	-	-	-	-
Chenopodio - stellarienea												
Crepis bursifolia L.	-	1	1	1	1	+	+	+	+	3	1	1
Crepis foetida L.	-	-	-	-	-	-	-	-	-	-	-	-
Crepis sancta (L.) Bornm.	-	+	+	+	+	-	+	+	+	2	2	1
Erigeron canadensis L.	-	-	-	-	-	-	-	-	-	-	-	-
Erigeron sumatrensis Retz.	-	2	3	3	3	3	3	3	+	+	+	+
Fumaria capreolata L. subsp. *capreolata*	-	-	-	-	-	-	-	-	-	-	-	-
Gastridium ventricosum (Gouan) Schinz & Thell.	-	-	-	-	-	-	-	-	-	-	-	-
Lactuca sativa L.	-	-	+	+	+	-	-	-	+	1	+	1
Medicago polymorpha L.	+	2	+	-	-	-	-	+	+	+	+	+
Senecio vulgaris L.	-	-	-	-	-	-	-	+	+	+	+	+
Solanum nigrum L.	-	-	-	-	-	-	-	+	+	+	+	+
Stellaria media (L.) Vill.	-	-	-	-	-	-	-	-	-	-	-	-
Trigonella italica (L.) Coulot & Rabaute	+	1	1	1	1	1	1	+	+	+	+	1
Parietarietea judaicae												
Cymbalaria muralis G.Gaertn., B.Mey. & Scherb.	-	-	-	-	-	-	-	-	-	-	-	-
Erigeron karvinskianus DC.	3	2	1	1	1	+	+	+	1	+	+	+
Sonchus tenerrimus L.	-	-	-	-	-	-	-	1	1	2	1	1
Reichardia picroides (L.) Roth	-	-	-	-	-	-	-	-	-	-	-	-
Polygono arenastri - Poetea annuae												
Euphorbia maculata L.	-	-	-	-	-	-	+	+	+	-	-	-
Euphorbia prostrata Aiton	-	-	-	-	-	-	+	+	+	+	+	+
Oxalis corniculata L.	-	-	-	-	-	-	+	+	+	+	-	-
Poa annua L.	-	-	-	-	-	-	-	-	-	-	-	-
Portulaca oleracea L.	-	-	-	-	-	-	-	-	-	-	-	-
Tuberarietea guttate												
Eleusine indica (L.) Gaertn.	-	-	-	-	-	-	-	-	-	-	-	-
Hypochaeris achyrophorus L.	-	+	+	+	+	+	+	+	+	+	1	1
Lotus ornithopodioides L.	-	-	-	-	-	-	-	-	-	-	-	-
Agrostietea stoloniferae												
Mentha spicata L.	-	-	-	-	-	-	-	-	-	-	-	-
Potentilla reptans L.	-	-	-	-	-	-	-	-	-	-	-	-
Molinio - Arrhenatheretea												
Cynodon dactylon (L.) Pers.	-	-	-	+	-	-	-	-	-	-	-	-
Lolium perenne L.	-	-	-	-	-	-	-	-	-	-	-	-
Saginetea maritimae												
Catapodium rigidum (L.) C.E.Hubb.	-	-	-	-	-	-	-	-	-	-	-	-
Hypochaeris glabra L.	-	-	-	-	-	-	-	-	-	-	-	-
Thlaspietea rotundifolii												
Dittrichia viscosa (L.) Greuter	-	-	-	-	-	-	-	-	-	+	+	+
Micromeria graeca (L.) Benth. ex Rchb.	-	-	-	-	-	-	-	-	-	-	-	+
Trifolio medii - Geranietea sanguinei												
Brachypodium rupestre (Host) Roem. & Schult.	-	-	-	-	-	-	-	-	-	-	-	-
Clinopodium nepeta (L.) Kuntze subsp. *nepeta*	-	-	-	-	-	-	-	-	-	-	-	-
Anomodonto - Polypodietea cambrici												
Saxifraga adscendens L.	-	-	-	-	-	-	-	-	-	-	-	-
Asplenietea trichomanis												
Lamium amplexicaule L.	-	-	-	-	-	-	-	-	-	-	-	-
Bidentetea tripartitae												
Echinochloa crus-galli (L.) P.Beauv. subsp. crus-galli	-	-	-	-	-	-	-	-	-	-	-	+
Cardaminetea hirsutae												
Campanula erinus L.	-	-	-	-	-	-	-	-	-	-	-	-
Galio aparines - urticetea dioicae												
Bituminaria bituminosa (L.) C.H.Stirt.	-	-	-	-	-	-	-	-	-	-	-	-
Isoeto-nano juncetae												
Blackstonia perfoliata (L.) Huds.	-	-	-	-	-	-	-	-	-	-	-	-
Koelerio glaucae - Corynephoretea canescentis												
Hypochaeris radicata L.	-	-	-	-	-	-	-	-	-	-	-	-
Rhamno catharticae - Prunetea spinosae												
Rosa canina L.	-	-	-	-	-	-	-	-	-	-	-	-
Rubus ulmifolius Schott	-	-	-	-	-	-	-	-	-	-	-	-
Salici purpureae - Populetea nigrae												
Platanus hispanica Mill. ex Münchh.	+	+	+	-	-	-	-	-	-	-	-	-
Sedo albi - Sclerentetea biennis												
Medicago minima (L.) L.	-	-	-	-	-	-	-	-	-	-	-	-

Supplementary Table S15. Vegetation surveys in the raised garden bed planter 4 (C4).

Species	Mar 21	Apr 21	May 21	Jun 21	Jul 21	Aug 21	Sep 21	Oct 21	Nov 21	Dec 21	Jan 22	Feb 22
Stellarienea mediae												
Andryala integrifolia L.	-	+	+	+	+	+	+	+	+	+	+	+
Digitaria sanguinalis (L.) Scop.	-	-	-	-	-	-	-	-	-	-	-	-
Fallopia convolvulus (L.) Á.Löve	-	-	-	-	-	-	+	+	+	+	+	+
Fumaria officinalis L.	-	-	-	-	-	-	-	-	-	-	-	+
Helminthotheca echioides (L.) Holub	+	-	-	-	-	-	-	-	-	-	-	-
Lathyrus tuberosus L.	-	-	-	-	-	-	-	-	-	-	-	-
Mercurialis annua L.	-	-	-	-	-	-	-	-	-	-	-	-
Nigella damascena L.	-	-	-	-	-	-	-	-	-	-	-	-
Orlaya grandiflora (L.) Hoffm.	-	-	-	-	-	-	-	-	-	-	-	-
Setaria pumila (Poir.) Roem. & Schult.	-	-	-	-	-	-	-	-	-	-	-	-
Sonchus asper (L.) Hill	-	-	+	+	-	-	-	-	-	-	-	-
Sonchus oleraceus L.	+	2	2	2	+	+	+	+	+	+	+	+
Veronica persica Poir.	-	-	-	-	-	-	-	-	-	-	-	-
Chenopodio - stellarienea												
Crepis bursifolia L.	-	1	1	1	1	-	-	+	+	+	1	1
Crepis foetida L.	-	-	-	-	-	-	-	-	-	-	-	-
Crepis sancta (L.) Bornm.	-	-	-	-	-	-	-	-	-	+	+	+
Erigeron canadensis L.	-	-	-	-	-	-	-	-	-	-	-	-
Erigeron sumatrensis Retz.	-	3	3	3	3	3	3	3	+	+	+	+
Fumaria capreolata L. subsp. *capreolata*	-	-	-	-	-	-	-	-	-	-	-	-
Gastridium ventricosum (Gouan) Schinz & Thell.	-	-	-	-	-	-	-	-	-	-	-	-
Lactuca sativa L.	-	+	+	+	+	-	-	-	-	-	+	+
Medicago polymorpha L.	-	-	1	1	+	+	+	+	2	2	2	3
Senecio vulgaris L.	-	-	-	-	-	-	-	-	-	-	-	+
Solanum nigrum L.	-	-	-	-	-	-	-	-	-	-	-	-
Stellaria media (L.) Vill.	-	-	-	-	-	-	-	-	-	-	-	-
Trigonella italica (L.) Coulot & Rabaute	-	2	+	+	-	-	-	+	+	1	1	2
Parietarietea judaicae												
Cymbalaria muralis G.Gaertn., B.Mey. & Scherb.	-	-	-	-	-	-	-	-	-	-	-	-
Erigeron karvinskianus DC.	2	1	+	+	+	+	+	+	+	+	+	+
Sonchus tenerrimus L.	-	-	-	-	-	-	-	+	+	+	+	+
Reichardia picroides (L.) Roth	-	-	-	-	-	-	-	-	-	-	-	-
Polygono arenastri - Poetea annuae												
Euphorbia maculata L.	-	-	-	-	-	-	+	+	+	+	+	-
Euphorbia prostrata Aiton	-	-	-	-	-	-	+	+	+	+	+	-
Oxalis corniculata L.	-	-	-	-	-	-	-	-	-	-	-	-
Poa annua L.	-	-	-	-	-	-	-	-	-	-	-	+
Portulaca oleracea L.	-	-	-	-	-	-	-	-	-	-	-	-
Tuberarietea guttate												
Eleusine indica (L.) Gaertn.	-	-	-	-	-	-	-	-	-	-	-	-
Hypochaeris achyrophorus L.	+	+	-	-	-	-	-	-	+	+	1	1
Lotus ornithopodioides L.	-	-	-	-	-	-	-	-	-	-	-	-
Agrostietea stoloniferae												
Mentha spicata L.	-	-	-	-	-	-	-	-	-	-	-	-
Potentilla reptans L.	-	-	-	-	-	-	-	-	-	-	-	-
Molinio - Arrhenatheretea												
Cynodon dactylon (L.) Pers.	-	-	-	-	-	-	-	-	-	-	1	+
Lolium perenne L.	-	-	-	-	-	-	-	-	-	-	-	-
Saginetea maritimae												
Catapodium rigidum (L.) C.E.Hubb.	-	-	-	-	-	-	-	-	-	-	-	-
Hypochaeris glabra L.	-	-	-	-	-	-	-	-	-	-	-	-
Thlaspietea rotundifolii												
Dittrichia viscosa (L.) Greuter	-	-	-	-	-	-	-	-	-	-	-	-
Micromeria graeca (L.) Benth. ex Rchb.	-	-	-	-	-	-	-	-	-	-	-	-
Trifolio medii - Geranietea sanguinei												
Brachypodium rupestre (Host) Roem. & Schult.	-	-	-	-	-	-	-	-	-	-	-	-
Clinopodium nepeta (L.) Kuntze subsp. *nepeta*	-	-	-	-	-	-	-	-	-	-	-	-
Anomodonto - Polypodietea cambrici												
Saxifraga adscendens L.	-	-	-	-	-	-	-	-	-	-	-	-
Asplenietea trichomanis												
Lamium amplexicaule L.	-	-	-	-	-	-	-	-	-	-	-	-
Bidentetea tripartitae												
Echinochloa crus-galli (L.) P.Beauv. subsp. crus-galli	-	-	-	-	-	-	-	-	-	-	-	-
Cardaminetea hirsutae												
Campanula erinus L.	-	-	-	-	-	-	-	-	-	-	-	-
Galio aparines - urticetea dioicae												
Bituminaria bituminosa (L.) C.H.Stirt.	-	-	-	-	-	-	-	-	-	-	-	-
Isoeto-nano juncetae												
Blackstonia perfoliata (L.) Huds.	-	-	-	-	-	-	-	-	-	-	-	-
Koelerio glaucae - Corynephoretea canescentis												
Hypochaeris radicata L.	-	-	-	+	-	-	-	-	-	-	-	-
Rhamno catharticae - Prunetea spinosae												
Rosa canina L.	-	-	-	-	-	-	-	-	-	-	-	-
Rubus ulmifolius Schott	-	-	-	-	-	-	-	-	-	-	-	-
Salici purpureae - Populetea nigrae												
Platanus hispanica Mill. ex Münchh.	-	-	-	-	-	-	-	-	-	-	-	-
Sedo albi - Sclerentetea biennis												
Medicago minima (L.) L.	-	1	+	+	-	-	-	-	-	-	-	-

Supplementary Table S16. Vegetation surveys in the raised garden bed planter 5 (C5).

Species	Mar 21	Apr 21	May 21	Jun 21	Jul 21	Aug 21	Sep 21	Oct 21	Nov 21	Dec 21	Jan 22	Feb 22
Stellarienea mediae												
Andryala integrifolia L.	-	+	+	-	-	-	-	-	-	-	-	-
Digitaria sanguinalis (L.) Scop.	-	-	-	-	-	-	-	-	-	-	+	+
Fallopia convolvulus (L.) Á.Löve	-	-	-	-	-	-	-	-	-	-	-	-
Fumaria officinalis L.	-	-	-	-	-	-	-	-	-	-	-	-
Helminthotheca echioides (L.) Holub	-	-	-	-	-	-	-	-	-	-	-	-
Lathyrus tuberosus L.	-	-	-	-	-	-	-	-	-	-	-	-
Mercurialis annua L.	-	-	-	-	-	-	-	-	-	-	-	-
Nigella damascena L.	-	-	-	-	-	-	-	-	-	-	-	-
Orlaya grandiflora (L.) Hoffm.	-	-	-	-	-	-	-	-	-	-	-	-
Setaria pumila (Poir.) Roem. & Schult.	-	-	-	-	-	-	-	-	-	-	-	-
Sonchus asper (L.) Hill	-	-	-	-	-	+	+	+	-	-	-	-
Sonchus oleraceus L.	-	+	+	+	-	+	+	+	-	-	+	+
Veronica persica Poir.	-	-	+	-	-	-	-	-	-	-	-	-
Chenopodio - stellarienea												
Crepis bursifolia L.	-	-	-	-	-	-	-	-	-	-	-	-
Crepis foetida L.	-	-	-	-	-	-	-	-	-	-	-	-
Crepis sancta (L.) Bornm.	-	+	+	+	+	-	-	-	-	-	+	+
Erigeron canadensis L.	-	-	-	-	-	-	-	-	-	-	-	-
Erigeron sumatrensis Retz.	-	1	1	2	2	2	2	+	+	+	+	+
Fumaria capreolata L. subsp. *capreolata*	-	-	-	-	-	-	-	-	-	-	-	-
Gastridium ventricosum (Gouan) Schinz & Thell.	-	-	-	-	-	-	-	-	-	-	-	-
Lactuca sativa L.	+	+	+	1	1	-	-	-	-	-	-	-
Medicago polymorpha L.	-	-	-	-	-	-	-	-	-	-	+	-
Senecio vulgaris L.	-	-	-	-	-	-	-	-	-	-	-	-
Solanum nigrum L.	-	+	+	+	+	+	+	-	-	-	-	-
Stellaria media (L.) Vill.	-	-	-	-	-	-	-	-	-	-	-	-
Trigonella italica (L.) Coulot & Rabaute	+	2	2	2	-	-	-	+	+	1	1	1
Parietarietea judaicae												
Cymbalaria muralis G.Gaertn., B.Mey. & Scherb.	-	+	-	-	-	-	-	-	-	-	-	-
Erigeron karvinskianus DC.	+	+	-	-	-	-	-	-	-	-	+	+
Sonchus tenerrimus L.	+	+	+	+	-	-	-	-	+	+	+	+
Reichardia picroides (L.) Roth	-	-	-	-	-	-	-	-	-	-	-	-
Polygono arenastri - Poetea annuae												
Euphorbia maculata L.	-	-	-	-	+	+	+	+	+	+	+	-
Euphorbia prostrata Aiton	-	-	-	+	-	-	+	+	+	+	+	-
Oxalis corniculata L.	-	-	-	-	-	-	-	-	-	-	-	-
Poa annua L.	-	-	-	-	-	-	-	-	-	-	-	-
Portulaca oleracea L.	-	-	-	-	-	-	-	-	-	-	-	-
Tuberarietea guttate												
Eleusine indica (L.) Gaertn.	-	-	-	-	-	-	-	-	-	-	-	-
Hypochaeris achyrophorus L.	+	+	+	+	+	+	-	-	-	-	+	1
Lotus ornithopodioides L.	-	-	-	-	-	-	-	-	-	-	-	-
Agrostietea stoloniferae												
Mentha spicata L.	-	-	-	-	-	-	-	-	-	-	-	-
Potentilla reptans L.	-	-	-	-	-	-	-	-	-	-	-	-
Molinio - Arrhenatheretea												
Cynodon dactylon (L.) Pers.	-	-	-	+	-	-	-	-	-	-	+	+
Lolium perenne L.	-	-	-	-	-	-	-	-	-	-	-	-
Saginetea maritimae												
Catapodium rigidum (L.) C.E.Hubb.	-	-	-	-	-	-	-	-	-	-	-	-
Hypochaeris glabra L.	-	-	-	-	-	-	-	-	-	-	-	-
Thlaspietea rotundifolii												
Dittrichia viscosa (L.) Greuter	-	-	-	-	-	-	-	-	-	-	-	-
Micromeria graeca (L.) Benth. ex Rchb.	-	-	-	-	-	-	-	-	-	-	-	+
Trifolio medii - Geranietea sanguinei												
Brachypodium rupestre (Host) Roem. & Schult.	-	-	-	-	-	-	-	-	-	-	-	-
Clinopodium nepeta (L.) Kuntze subsp. *nepeta*	+	+	+	+	+	+	+	+	+	+	+	+
Anomodonto - Polypodietea cambrici												
Saxifraga adscendens L.	-	-	-	-	-	-	-	-	-	-	-	-
Asplenietea trichomanis												
Lamium amplexicaule L.	-	-	-	-	-	-	-	-	-	-	-	-
Bidentetea tripartitae												
Echinochloa crus-galli (L.) P.Beauv. subsp. crus-galli	-	-	-	-	-	-	-	-	-	-	-	+
Cardaminetea hirsutae												
Campanula erinus L.	-	-	-	-	-	-	-	-	-	-	-	-
Galio aparines - urticetea dioicae												
Bituminaria bituminosa (L.) C.H.Stirt.	-	-	-	-	-	-	-	-	-	-	-	-
Isoeto-nano juncetae												
Blackstonia perfoliata (L.) Huds.	-	-	-	-	-	-	-	-	-	-	-	-
Koelerio glaucae - Corynephoretea canescentis												
Hypochaeris radicata L.	-	-	-	-	+	+	-	-	-	-	-	-
Rhamno catharticae - Prunetea spinosae												
Rosa canina L.	-	-	-	-	-	-	-	-	-	-	-	-
Rubus ulmifolius Schott	-	-	-	-	-	-	-	-	-	-	-	-
Salici purpureae - Populetea nigrae												
Platanus hispanica Mill. ex Münchh.	-	-	-	-	-	-	-	-	-	-	-	-
Sedo albi - Sclerentetea biennis												
Medicago minima (L.) L.	-	-	-	-	-	-	-	+	+	+	+	-

Supplementary Table S17. Vegetation surveys in the raised garden bed planter 6 (C6).

Species	Mar 21	Apr 21	May 21	Jun 21	Jul 21	Aug 21	Sep 21	Oct 21	Nov 21	Dec 21	Jan 22	Feb 22
Stellarienea mediae												
Andryala integrifolia L.	-	-	-	-	-	-	-	-	-	-	-	-
Digitaria sanguinalis (L.) Scop.	-	-	-	-	-	-	-	-	-	-	+	+
Fallopia convolvulus (L.) Á.Löve	-	-	-	-	-	-	-	-	-	-	-	-
Fumaria officinalis L.	-	-	-	-	-	-	-	-	-	-	-	-
Helminthotheca echioides (L.) Holub	-	-	-	-	-	-	-	-	-	-	-	-
Lathyrus tuberosus L.	-	-	-	-	-	-	-	-	-	-	-	+
Mercurialis annua L.	-	-	-	-	-	-	-	-	-	-	-	-
Nigella damascena L.	-	-	-	-	-	-	-	-	-	-	-	-
Orlaya grandiflora (L.) Hoffm.	-	-	-	-	-	-	-	-	-	-	-	-
Setaria pumila (Poir.) Roem. & Schult.	-	-	-	-	-	-	-	-	-	-	-	-
Sonchus asper (L.) Hill	-	-	-	-	-	-	-	-	-	-	-	-
Sonchus oleraceus L.	-	+	+	+	-	-	-	-	-	-	-	-
Veronica persica Poir.	-	-	-	-	-	-	-	-	-	-	-	-
Chenopodio - stellarienea												
Crepis bursifolia L.	-	-	-	-	-	-	-	-	+	+	+	+
Crepis foetida L.	-	-	-	-	-	-	-	-	-	-	-	-
Crepis sancta (L.) Bornm.	-	-	+	+	+	-	-	-	-	-	-	-
Erigeron canadensis L.	-	-	-	-	-	-	-	-	-	-	-	-
Erigeron sumatrensis Retz.	-	-	-	-	-	-	-	-	-	-	-	-
Fumaria capreolata L. subsp. *capreolata*	-	-	-	-	-	-	-	-	-	-	-	-
Gastridium ventricosum (Gouan) Schinz & Thell.	-	-	-	-	-	-	-	-	-	-	-	-
Lactuca sativa L.	-	-	-	-	-	-	-	-	-	-	-	-
Medicago polymorpha L.	-	-	-	-	-	-	-	-	-	-	+	-
Senecio vulgaris L.	-	-	-	-	-	-	-	-	-	-	-	-
Solanum nigrum L.	-	-	-	-	-	-	-	-	-	-	-	-
Stellaria media (L.) Vill.	-	-	-	-	-	-	-	-	-	-	-	-
Trigonella italica (L.) Coulot & Rabaute	+	+	3	3	-	-	-	+	+	2	2	1
Parietarietea judaicae												
Cymbalaria muralis G.Gaertn., B.Mey. & Scherb.	-	-	-	-	-	-	-	-	-	-	-	-
Erigeron karvinskianus DC.	-	-	-	-	-	-	-	-	-	-	+	-
Sonchus tenerrimus L.	-	-	-	-	-	-	-	-	-	-	+	+
Reichardia picroides (L.) Roth	-	-	-	-	-	-	-	-	-	-	-	-
Polygono arenastri - Poetea annuae												
Euphorbia maculata L.	-	-	-	-	+	+	+	+	+	+	+	-
Euphorbia prostrata Aiton	-	-	-	+	-	-	+	+	+	+	+	+
Oxalis corniculata L.	-	-	-	-	-	-	-	-	-	-	-	-
Poa annua L.	-	-	-	-	-	-	-	-	-	-	-	-
Portulaca oleracea L.	-	-	-	-	-	-	-	-	-	-	-	-
Tuberarietea guttate												
Eleusine indica (L.) Gaertn.	-	-	-	-	-	-	-	-	-	-	-	-
Hypochaeris achyrophorus L.	-	-	-	-	-	-	-	-	-	-	-	+
Lotus ornithopodioides L.	-	-	-	-	-	-	-	-	-	-	-	-
Agrostietea stoloniferae	-	-	-	-	-	-	-	-	-	-	-	-
Mentha spicata L.	-	-	-	-	-	-	-	-	-	-	-	-
Potentilla reptans L.	-	-	-	-	-	-	-	-	-	-	-	-
Molinio - Arrhenatheretea												
Cynodon dactylon (L.) Pers.	-	-	-	+	-	-	-	-	-	-	+	+
Lolium perenne L.	-	-	-	-	-	-	-	-	-	-	-	-
Saginetea maritimae												
Catapodium rigidum (L.) C.E.Hubb.	-	-	-	-	-	-	-	-	-	-	-	-
Hypochaeris glabra L.	-	-	-	-	-	-	-	-	-	-	-	-
Thlaspietea rotundifolii												
Dittrichia viscosa (L.) Greuter	-	-	-	-	-	-	-	-	-	-	-	-
Micromeria graeca (L.) Benth. ex Rchb.	-	-	-	+	+	+	+	+	+	+	+	+
Trifolio medii - Geranietea sanguinei												
Brachypodium rupestre (Host) Roem. & Schult.	-	-	-	-	-	-	-	-	-	-	-	-
Clinopodium nepeta (L.) Kuntze subsp. *nepeta*	+	+	+	+	+	+	+	+	+	+	+	+
Anomodonto - Polypodietea cambrici												
Saxifraga adscendens L.	-	-	-	-	-	-	-	-	-	-	-	-
Asplenietea trichomanis												
Lamium amplexicaule L.	-	-	-	-	-	-	-	-	-	-	-	-
Bidentetea tripartitae												
Echinochloa crus-galli (L.) P.Beauv. subsp. crus-galli	-	-	-	-	-	-	-	-	-	-	-	+
Cardaminetea hirsutae												
Campanula erinus L.	-	-	-	-	-	-	-	-	-	-	-	-
Galio aparines - urticetea dioicae												
Bituminaria bituminosa (L.) C.H.Stirt.	-	-	-	-	-	-	-	-	-	-	-	-
Isoeto-nano juncetae												
Blackstonia perfoliata (L.) Huds.	-	-	-	-	-	-	-	-	-	-	-	-
Koelerio glaucae - Corynephoretea canescentis												
Hypochaeris radicata L.	-	-	-	-	-	+	-	-	-	-	-	-
Rhamno catharticae - Prunetea spinosae												
Rosa canina L.	-	-	-	-	-	-	-	-	-	-	-	-
Rubus ulmifolius Schott	-	-	-	-	-	-	-	-	-	-	-	-
Salici purpureae - Populetea nigrae												
Platanus hispanica Mill. ex Münchh.	-	-	-	-	-	-	-	-	-	-	-	-
Sedo albi - Sclerentetea biennis												
Medicago minima (L.) L.	-	2	2	2	-	-	-	-	-	-	-	-
